# Specificity and catalysis hardwired at the RNA–protein interface in a translational proofreading enzyme

**DOI:** 10.1038/ncomms8552

**Published:** 2015-06-26

**Authors:** Sadeem Ahmad, Sowndarya Muthukumar, Santosh Kumar Kuncha, Satya Brata Routh, Antony S.K. Yerabham, Tanweer Hussain, Venu Kamarthapu, Shobha P Kruparani, Rajan Sankaranarayanan

**Affiliations:** 1 Centre for Cellular and Molecular Biology, Council of Scientific and Industrial Research, Uppal Road, Hyderabad 500007, India

## Abstract

Proofreading modules of aminoacyl-tRNA synthetases are responsible for enforcing a high fidelity during translation of the genetic code. They use strategically positioned side chains for specifically targeting incorrect aminoacyl-tRNAs. Here, we show that a unique proofreading module possessing a D-aminoacyl-tRNA deacylase fold does not use side chains for imparting specificity or for catalysis, the two hallmark activities of enzymes. We show, using three distinct archaea, that a side-chain-stripped recognition site is fully capable of solving a subtle discrimination problem. While biochemical probing establishes that RNA plays the catalytic role, mechanistic insights from multiple high-resolution snapshots reveal that differential remodelling of the catalytic core at the RNA–peptide interface provides the determinants for correct proofreading activity. The functional crosstalk between RNA and protein elucidated here suggests how primordial enzyme functions could have emerged on RNA–peptide scaffolds before recruitment of specific side chains.

## Abstract (web summary)

The editing domain of aminoacyl-tRNA synthetases is responsible for removing non-cognate amino acids from mischarged tRNAs. Here the authors show that the D-aminoacyl-tRNA deacylase fold of archaeal ThrRS does not rely on protein side chains for substrate specificity and catalysis.

## Graphical abstract

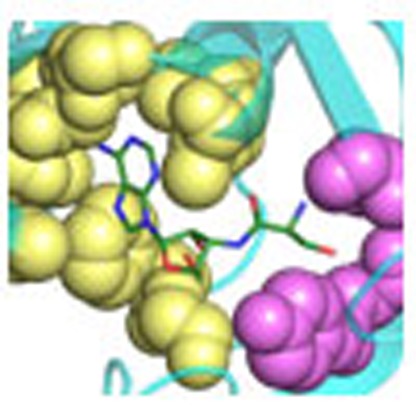

Protein enzymes strategically employ side chains in their active sites to provide functional groups for catalysis as well as substrate specificity, the two hallmarks of enzymatic activity. Crucial role played by side chains has been observed in several enzymatic systems with a classical text book case being that of serine proteases^1^. Aminoacyl-transfer RNA (tRNA) synthetases (aaRSs) are thought to be one of the first protein enzymes to have emerged from the RNA world^2^ and face some of the most difficult substrate recognition problems during translation of the genetic code^3^. It has been shown that nearly half of the 20 aaRSs misactivate a wrong amino acid on tRNAs and therefore a proofreading/editing function is associated with them to correct such errors^4^,^5^,^6^,^7^,^8^,^9^,^10^,^11^,^12^,^13^,^14^. Additional domains associated with aaRSs either in *cis* or in *trans* have been shown to be majorly responsible for proofreading activity. Similar to other known enzymatic systems, the proofreading enzymes have been shown to use side chains for their function^15^,^16^,^17^,^18^,^19^,^20^,^21^,^22^.

The proofreading modules are designed for solving intricate substrate discrimination problems, for example, in the case of isoleucyl-tRNA synthetase or threonyl-tRNA synthetase (ThrRS), a difference of a single methyl group between two large substrates Ile/Val- or Thr/Ser-tRNA. The presence of significantly higher excess of cognate aminoacyl-tRNA over the non-cognate one in the cellular scenario compounds the problem and therefore imposes a stricter constraint for the proofreading domains so as to avoid depleting the cognate aminoacyl-tRNA pool. This is particularly important considering that aminoacyl-tRNAs can be resampled by editing domains even after binding to elongation factor-Tu^23^. The active site in the proofreading enzymes is comprised of two pockets, that is, the adenosine pocket and the amino-acid pocket, which bind the terminal adenosine (A76) of tRNA and the non-cognate amino acid, respectively, separated by a distance of ∼10 Å (Fig. 1a). Extensive biochemical and structural studies on multiple proofreading modules have shown that the side chains in the amino-acid pocket are strategically positioned to either sterically or chemically reject the cognate substrate from the active site, thereby preventing its gratuitous deacylation^16^,^18^,^20^,^21^,^22^,^24^. Mutation of these crucial residues relaxes the specificity of these enzymes and leads to significant deacylation of the cognate aminoacyl-tRNA substrate (Supplementary Table 1).

We have been interested in a D-aminoacyl-tRNA deacylase (DTD) fold that is present across the domains of life in two different functional contexts: as an N-terminal editing domain (NTD) of archaeal ThrRS, which specifically removes non-cognate L-serine mischarged on tRNA^Thr^, and as a freestanding ‘chiral proofreading' enzyme, which removes D-amino acids from tRNAs in bacteria and eukaryotes. Both share a striking structural and mechanistic similarity highlighting a common ancestry^25^,^26^. DTD-fold's proposed role in perpetuation of homochirality, its association with translational apparatus and its universal distribution across the three domains of life strongly suggest a primordial origin for this fold. The earlier work on NTD from *Pyrococcus abyssi* (PabNTD) had indicated an RNA-assisted catalytic mechanism implicating the role of 2′-OH in activating a water molecule^26^. The presence or absence of this crucial water was implicated in serine/threonine discrimination by this enzyme^27^. Here we show, with the help of structural, biochemical and biophysical studies from three distant organisms, that the substrate specificity in this domain is determined by a differential remodelling of the catalytic centre at the RNA–protein interface rather than just the presence or absence of catalytic water as previously proposed^27^. While demonstrating that the tRNA provides the catalytic group, we show that the side chains within the recognition site are dispensable for the proofreading function, suggesting a primordial mode of protein- or peptide scaffold-based enzymatic activity evolved alongside that of RNA.

## Results

### Dispensability of side chains for proofreading activity

**Figure 1:**
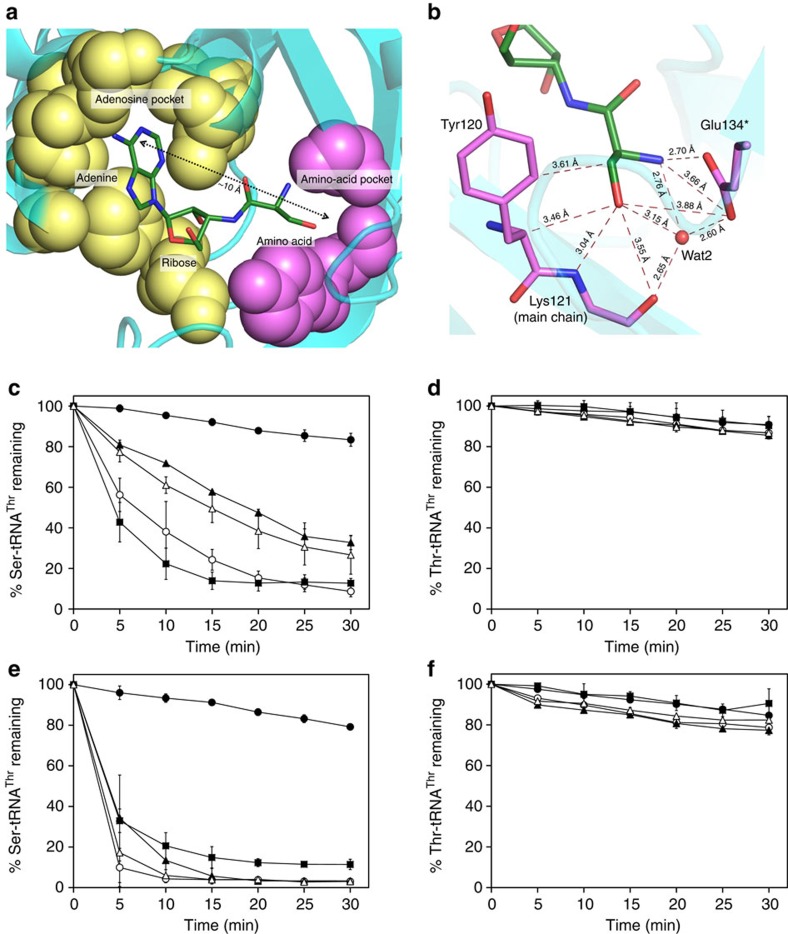
Dispensable role of side chains in substrate discrimination. (**a**) The two-pocket architecture of proofreading sites showing the adenosine pocket (yellow) and amino-acid pocket (pink) ∼10 Å apart. (**b**) The interaction network of the amino-acid moiety (serine) in the PabNTD pocket. (**c**) Deacylation of L-Ser-(Ec)tRNA^Thr^ and (**d**) L-Thr-(Ec)tRNA^Thr^ by buffer (closed circles), PabNTD wild type (open circle), Y120A (closed triangles), E134A (open triangles) and Y120A/E134A (closed squares). Deacylation of (**e**) L-Ser-(Ec)tRNA^Thr^ and (**f**) L-Thr-(Ec)tRNA^Thr^ by buffer (closed circles), ApNTD wild type (open circle), Y115A (closed triangles), E129A (open triangles) and Y115A/E129A (closed squares). The error bars indicate s.d. of three independent experiments.

The amino-acid pocket in PabNTD employs two side chains, that is, Tyr120 and Glu134, forming an extensive network of interactions with the serine moiety of the substrate (Fig. 1b). To determine the role of these residues in substrate specificity, we mutated these two residues to Ala and checked for their deacylation function against non-cognate L-Ser-(Ec)tRNA^Thr^ and cognate L-Thr-(Ec)tRNA^Thr^ (Ec denotes *Escherichia coli*). Surprisingly, both Y120A and E134A mutants were efficient in discriminating between serine and threonine substrates, since deacylation was observed only against L-Ser-(Ec)tRNA^Thr^ while no activity was found against L-Thr-(Ec)tRNA^Thr^ (Fig. 1c,d). We then generated a double-mutant Y120A/E134A, which was completely devoid of side chains in the amino-acid pocket. The enormous space created by shaving the side chains is expected to allow the extra γ-methyl group of threonine to be accommodated in the pocket without much difficulty. Strikingly, even the double-mutant Y120A/E134A, with the substrate-binding pocket totally stripped of the side chains, was remarkably efficient in cognate/non-cognate discrimination (Fig. 1c,d). This clearly shows that the determinants of substrate specificity in PabNTD do not rely on the side-chain atoms. It may be noted here that there was no significant spontaneous deacylation in the buffer for both L-Ser-(Ec)tRNA^Thr^ and L-Thr-(Ec)tRNA^Thr^ up to 30 min. We confirmed the observation by carrying out biochemical studies in a related organism *P. furiosus* (PfuNTD) also, sharing ∼80% identity with PabNTD, and the double-mutant Y120A/E134A in PfuNTD completely retained the substrate specificity like the wild-type enzyme (Supplementary Fig. 1).

**Figure 2:**
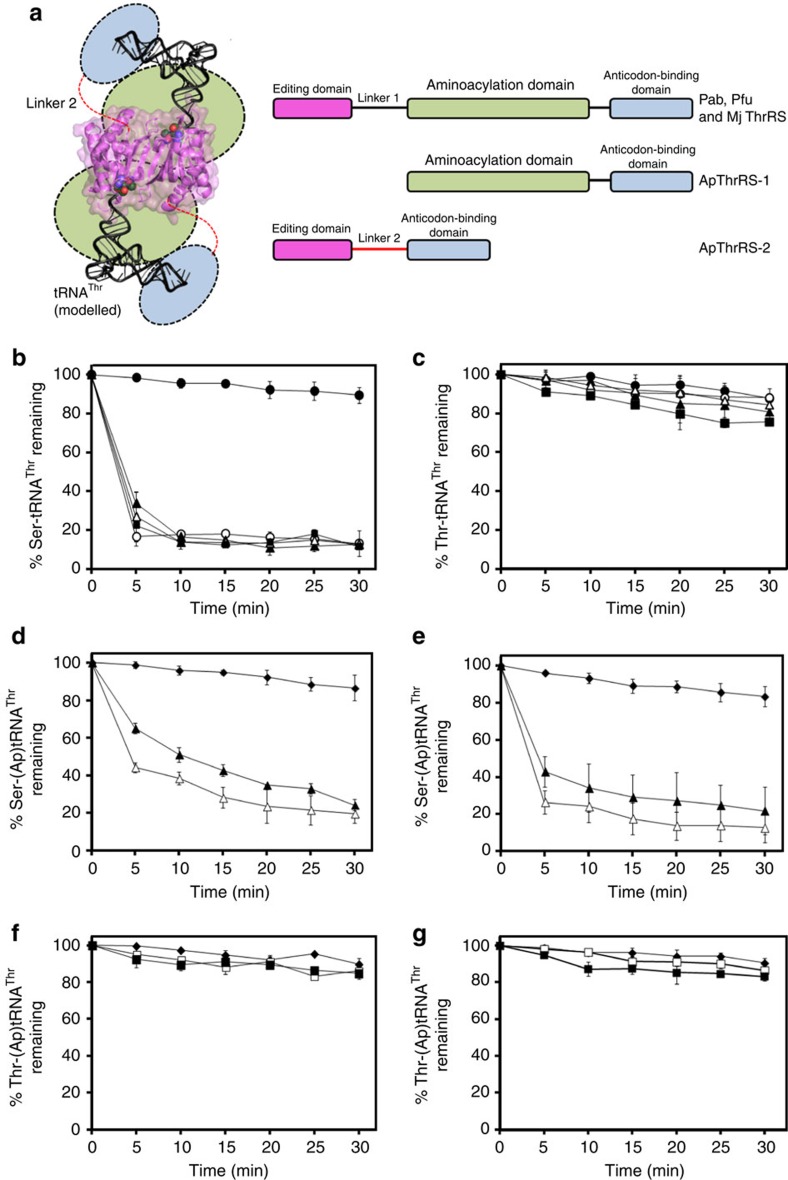
Domain organization of archaeal threonyl-tRNA synthetase (ThrRS) and deacylation activities of ApThrRS-2 and PabNTD. (**a**) NTD is either coupled to the aminoacylation domain as in Pab, Pfu and MjThrRSs or found as a separate protein linked to the anticodon-binding domain as in ApThrRS-2. The schematic on the left-hand side represents the structure of NTD in two different contexts. While the NTD dimer shown is the actual crystal structure, tRNAs are modelled and the aminoacylation domain, anticodon-binding domain and linker 2 are schematically represented. (**b**) Deacylation of L-Ser-(Ec)tRNA^Thr^ and (**c**) L-Thr-(Ec)tRNA^Thr^ by buffer (closed circles), ApThrRS-2 wild type (open circles), Y115A (closed triangles), E129A (open triangles) and Y115A/E129A (closed squares). (**d**) Deacylation of L-Ser-(Ap)tRNA^Thr^ with buffer (closed diamonds), 150 nM ApThrRS-2 wild type (open triangles) and 150 nM ApThrRS-2 Y115A/E129A (closed triangle). (**e**) Deacylation of L-Ser-(Ap)tRNA^Thr^ with buffer (closed diamonds), 150 nM PabNTD wild type (open triangles) and 150 nM PabNTD Y120A/E134A (closed triangles). (**f**) Deacylation of L-Thr-(Ap)tRNA^Thr^ with buffer (closed diamonds), 15 μM ApThrRS-2 wild type (open squares) and 15 μM ApThrRS-2 Y115A/E129A (closed squares). (**g**) Deacylation of L-Thr-(Ap)tRNA^Thr^ with buffer (closed diamonds), 15 μM PabNTD wild type (open squares) and 15 μM PabNTD Y120A/E134A (closed squares). The error bars indicate s.d. of three independent experiments.

To rule out any genus-related phenomenon and to test the universality of the observation, we chose to do biochemical experiments with NTD from *Aeropyrum pernix* (ApNTD), which belongs to a different phylum (*Crenarchaeota*), and shares ∼43% identity with PabNTD. Moreover, ApNTD is a *trans* editing domain as opposed to PabNTD, which is a *cis* module. Remarkably, the corresponding mutants Y115A, E129A and Y115A/E129A in ApNTD also retained completely the capability to discriminate between seryl and threonyl substrates (Fig. 1e,f). We also carried out the deacylation studies with full-length ThrRS-2 from *A. pernix* (ApThrRS-2), which has NTD linked to the anticodon-binding domain (Fig. 2a), to ensure that the observations with NTDs are not due to artefacts arising from domain isolation. The mutants of ApThrRS-2 also showed no deacylation of L-Thr-(Ec)tRNA^Thr^ while retaining activity against L-Ser-(Ec)tRNA^Thr^ (Fig. 2b,c), thereby clearly showing that the side-chain dispensability for substrate specificity in this enzyme is biologically relevant.

To rule out any artefacts due to the use of heterologous enzyme–tRNA systems, that is, archaeal enzyme and bacterial tRNA in our biochemical assays, we generated cognate and non-cognate substrates using tRNA^Thr^ from *A. pernix* to test the archaeal enzymes. ApThrRS-2 wild type and Y115A/E129A (each at 150 nM concentration) showed similar activity on L-Ser-(Ap)tRNA^Thr^ (Ap denotes *A. pernix*) (Fig. 2d). PabNTD wild type and Y120A/E134A also showed the same trend in activity when tested with L-Ser-(Ap)tRNA^Thr^ (Fig. 2e). Hence, experiments carried out using archaeal enzymes and archaeal tRNA validated our conclusions derived from using archaeal enzymes and bacterial tRNA. It is worthwhile mentioning that even a 100-fold increase in enzyme concentration of either ApThrRS-2 wild type or double mutant, failed to deacylate the cognate substrate, L-Thr-(Ap)tRNA^Thr^ (Fig. 2f); the same was true in the case of PabNTD as well (Fig. 2g). This demonstrated that substrate specificity and discriminatory efficiency of the proofreading domains are maintained in the wild type as well as the mutants. While the experiments were performed with unmodified tRNA from archaea, the fact that there are no modifications of the bases reported at the acceptor stem of bacterial and archaeal tRNA^Thr^ (refs 28, 29) suggests that the modifications are not involved in discrimination.

The observed first-order rate constant, *k*_obs_, under single-turnover conditions, for different enzyme–substrate pairs was also calculated to determine the differences in the reaction rates (Table 1; Supplementary Fig. 2). The *k*_obs_ for deacylation of L-Ser-(Ap)tRNA^Thr^ by ApThrRS-2 wild type was 0.352±0.060 min^−1^, whereas that by ApThrRS-2 Y115A/E129A was 0.213±0.022 min^−1^. PabNTD wild-type deacylated L-Ser-(Ap)tRNA^Thr^ with a *k*_obs_ value of 0.209±0.026 min^−1^, while PabNTD Y120A/E134A catalysed the same reaction with a *k*_obs_ value of 0.143±0.018 min^−1^. Spontaneous deacylation of the substrate occurred at a rate of 0.004±0.0004, min^−1^, which was negligible when compared with enzyme-catalysed reactions. Thus, there was only ∼1.7-fold and ∼1.5-fold reduction in the rate of catalysis by ApThrRS-2 Y115A/E129A and PabNTD Y120A/E134A when compared with their respective wild-type enzymes. Moreover, the activity of the enzymes (wild type and double mutant) on the cognate substrate, L-Thr-(Ap)tRNA^Thr^, was similar to spontaneous deacylation of the substrate. It is, therefore, evident from analysis of single-turnover kinetics of these enzymes that the active-site mutants retain their capability of substrate discrimination and catalyse the deacylation reaction like the wild-type enzyme, albeit at a slightly reduced rate.

It is very interesting to note here that completely conserved residues found in a crucial region of the active site and involved in a number of interactions with the substrate do not play any role in substrate discrimination (Fig. 1b). Significantly, the recalcitrance of NTD mutants to cognate substrate deacylation demonstrates an unprecedented mechanism of specificity, wherein a subtle discrimination of a single methyl group is performed without the role of side chains in the pocket.

### Structural basis of binding cognate vs non-cognate substrate

To understand this intriguing mechanism of cognate substrate rejection in NTD, we have solved high-resolution crystal structures of ApNTD, as well as NTD from *Methanococcus jannaschii* (MjNTD) in complex with non-cognate and cognate substrate analogues L-seryl-3′-aminoadenosine (L-Ser3AA) and L-threonyl-3′-aminoadenosine (L-Thr3AA), respectively (Supplementary Table 2). The co-crystal structures of ApNTD were solved in *P*4_1_2_1_2 space group with only one monomer in the asymmetric unit, as the biological dimer axis coincided with the crystallographic twofold axis (Supplementary Fig. 3). In the case of MjNTD, L-Ser3AA and L-Thr3AA complex were crystallized in *C*2 and *P*2_1_ space groups with six and four monomers, respectively, in the asymmetric unit corresponding to three and two dimers. However, a clear and complete ligand density was observed in only chain A in the case of MjNTD co-crystals and therefore only this monomer was used for analysis. The possible reason for this could be the remarkably low binding affinity for the ligand in MjNTD, as revealed by binding studies later in this section. The structures solved in this study, together with the available structural data on PabNTD, provide us with independent observations from three different organisms. Earlier work on PabNTD had proposed a cognate substrate rejection model, wherein the accommodation of extra γ-methyl group upon threonine binding leads to an outward movement of Tyr120, and a concomitant inward movement of the adjacent Lys121 sterically excludes the crucial catalytic water Wat1 from the reaction site^27^ (Supplementary Fig. 4a). Being the only report till date where even the cognate substrate has been shown to bind in a proofreading pocket, the study underlined the key role of functional positioning of the substrate in determining enzyme specificity. However, if we compare the non-cognate and cognate substrate-bound structures of ApNTD and MjNTD and put them in perspective with PabNTD, there are two very striking observations. First, the rejection of threonine from the pocket does not depend on Tyr120 repositioning as earlier proposed, since the corresponding residue Tyr115 shows no significant movement in the case of ApNTD (Supplementary Fig. 4b). Second, and more importantly, just the presence or absence of the water Wat1 is not sufficient to determine whether the substrate would be deacylated by the enzyme or not, since Wat1 is also present in the MjNTD+L-Thr3AA structure (Supplementary Fig. 4c). We also performed biochemical studies with MjNTD against L-Ser-(Ec)tRNA^Thr^ and L-(Ec)Thr-tRNA^Thr^ to ensure that this enzyme is efficient in non-cognate/cognate discrimination. There was a clear activity against L-Ser-(Ec)tRNA^Thr^ while no deacylation was observed against L-Thr-(Ec)tRNA^Thr^ even though Wat1 was present in both L-Ser3AA- and L-Thr3AA-bound structures (Supplementary Fig. 5). The lack of activity against L-Thr-(Ec)tRNA^Thr^ by MjNTD strongly suggests a role of other determinants besides the presence/absence of Wat1 in substrate discrimination. Interestingly, the mutation of Glu135 to Ala in MjNTD led to a loss of activity against L-Ser-(Ec)tRNA^Thr^, while Y121A still retained deacylation activity suggesting a role for Glu135 side chain in this homologue (Supplementary Fig. 5a). However, more importantly, since no deacylation activity was observed against L-Thr-(Ec)tRNA^Thr^ in any of these mutants, their role in substrate discrimination can be ruled out (Supplementary Fig. 5b).

### Universality of cognate binding without catalysis

**Figure 3:**
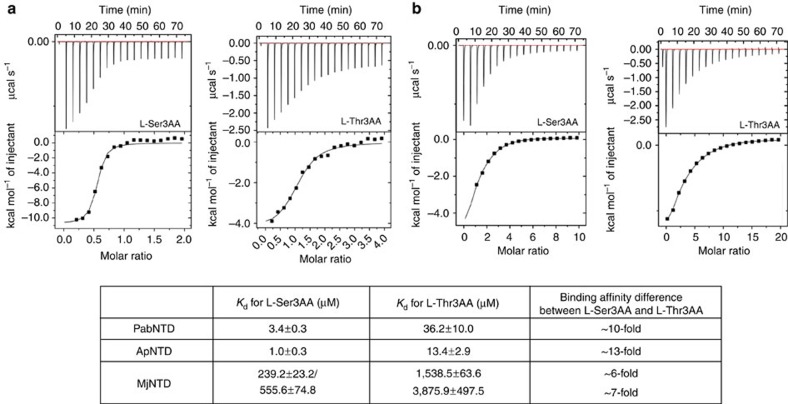
Substrate analogue binding in NTD. Binding studies with (**a**) ApNTD and (**b**) MjNTD. The binding parameters for PabNTD have been taken from a previous study^27^. One-binding-site mode was used for both ApNTD and PabNTD while a two-binding-site mode was used in the case of MjNTD for analysis.

We further carried out isothermal titration calorimetry (ITC)-based binding studies to see whether the discrimination can be accounted for by affinity difference between non-cognate and cognate substrates. However, ApNTD and MjNTD showed an affinity difference of ∼13-fold and 7-fold, respectively, between L-Ser3AA and L-Thr3AA (Fig. 3). These affinity differences are too small to completely account for substrate discrimination just as in the case of PabNTD where only a 10-fold affinity difference was observed between L-Ser3AA and L-Thr3AA^27^, especially considering the presence of real excess of cognate aminoacyl-tRNA than the non-cognate one in the cellular scenario. This clearly suggests that the mechanism of substrate specificity in NTD is largely independent of binding affinity difference. Moreover, ITC data as well as structural studies confirm that cognate substrate binding observed in PabNTD earlier^27^ is a universal phenomenon in this family of enzymes, irrespective of organisms.

### Plasticity in substrate recognition mode by NTDs

**Figure 4:**
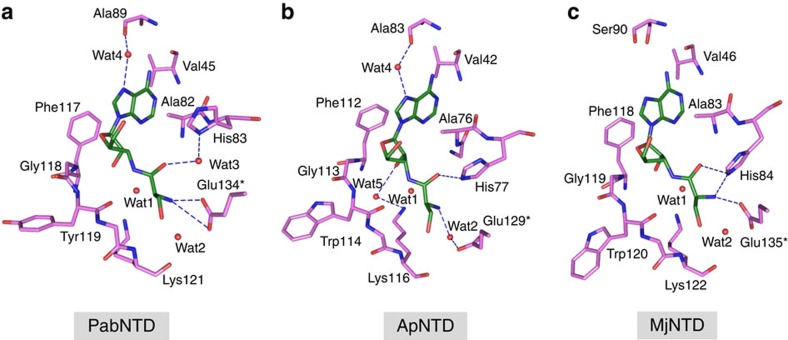
The plasticity in substrate recognition modes in NTD. The varying interactions of the ligand L-Ser3AA (green) in (**a**) PabNTD, (**b**) ApNTD and (**c**) MjNTD are shown as blue dashes.

Interestingly, while there is strict conservation in the adenosine binding mode in all three NTDs, they display a remarkable plasticity in their amino-acid recognition modes (Fig. 4). The carbonyl oxygen of the substrate has a water-mediated interaction with His83 in PabNTD, while the same atom interacts directly with the corresponding His77 in ApNTD and His84 in MjNTD. The α-NH_2_ group of the substrate is held by a cross-subunit interaction with the two carboxylate oxygens of Glu134 from the other monomer in PabNTD. However, in ApNTD, it has only a water-mediated interaction with the corresponding Glu129, whereas in the case of MjNTD, this group is directly recognized by both Glu135 and His84. Moreover, the catalytic sites in PabNTD and MjNTD accommodate a single water molecule, which is the putative catalytic water Wat1. In ApNTD, on the other hand, the extra space in the vicinity of Wat1 allows a second ApNTD-specific water Wat5 to be accommodated. These striking variations among the three NTDs not only highlight an inherent plasticity in the substrate-binding modes in this enzyme family but also present a rare paradigm in enzymology, wherein an invariant active site performing identical biochemical function recognizes the same substrate with significant differences in the interaction network.

### Mechanistic basis for discrimination

**Figure 5:**
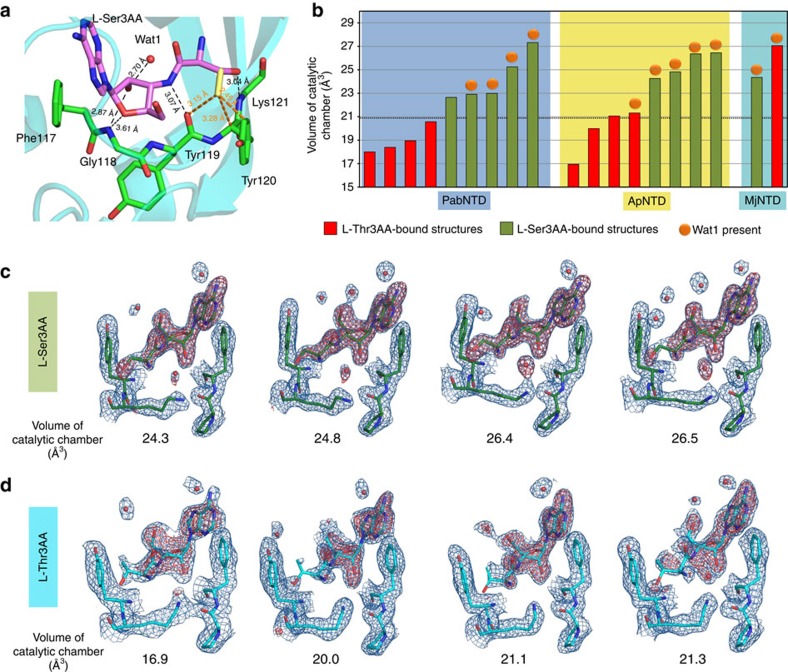
Role of the RNA–protein interface in substrate specificity. (**a**) An extra methyl group on the Cβ of the seryl moiety of the substrate would have clashes with the main-chain atoms lining the RNA–protein interface. (**b**) The volume of the catalytic chamber (the space in which Wat1 is positioned) in different ligand-bound crystal structures of PabNTD, ApNTD and MjNTD. A circle on top of the bar indicates the presence of Wat1. The threshold volume is ∼21 Å^3^, below which the water molecule cannot be accommodated. All L-Ser3AA-bound structures are above this threshold. All L-Thr3AA-bound structures in PabNTD fall below this mark, whereas one observation in ApNTD is slightly above and the only observation in MjNTD is significantly above the threshold volume. The unbiased maps 2*F*_o_−*F*_c_ (blue) and *F*_o_−*F*_c_ (red) for ligand and Wat1 from multiple crystal structures of ApNTD with (**c**) L-Ser3AA and (**d**) L-Thr3AA arranged in increasing order of the volume of the catalytic chamber. The 2*F*_o_−*F*_c_ maps are contoured at 1*σ* for L-Ser3AA complexes and 0.8*σ* for L-Thr3AA complexes. All *F*_o_−*F*_c_ maps are contoured at 3*σ*. The seryl moiety and the Wat1 show a clear electron density in all L-Ser3AA-bound structures, whereas the density for the threonyl moiety becomes weaker as the volume of the catalytic chamber increases in all L-Thr3AA-bound structures.

To understand the mechanistic basis of serine/threonine discrimination, the structural data from PabNTD, ApNTD and MjNTD were intricately analysed. L-Ser3AA tightly binds in the active-site pocket in such a way that a conserved hydrogen bond with the main-chain N atom of Lys121 (residue number corresponding to PabNTD) fixes the β-OH group of the substrate in all three NTDs. With this mode of binding, an extra methyl group on the Cβ of the substrate would have serious clashes with not only Tyr120 side-chain and main-chain atoms as noted earlier but also with the main-chain O of Tyr119 (Trp114 and Trp120 in ApNTD and MjNTD, respectively) (Fig. 5a). In our earlier study, we had mainly focused on the clash with Tyr120 side chain as it was not only the most striking one but also there was a significant movement of this side chain upon L-Thr3AA binding^27^. This unfavourable environment for the γ-methyl group would perturb the RNA–protein interface between the terminal adenosine of tRNA and the residues from 117 to 120 (numbers correspond to PabNTD) in the active site (Fig. 5a). In PabNTD, this perturbation leads to constriction of the space available in the catalytic chamber formed by the RNA–protein interface, leading to steric exclusion of the putative catalytic water (Fig. 5b). On the other hand, in ApNTD and MjNTD it leads to a loosening of interactions of the threonyl moiety of the substrate in the pocket, which would not allow it to be fixed with sufficient rigidity for the nucleophilic attack to take place (Supplementary Fig. 6). While this loosening of interactions is very striking in the case of MjNTD, in ApNTD there is a subtle relaxation. Therefore, to ensure that the loosening of interactions observed in ApNTD is significant, we solved multiple high-resolution crystal structures of this enzyme with L-Ser3AA and L-Thr3AA to obtain random snapshots of the substrate analogue in the active site (Supplementary Tables 3 and 4). The average interaction distances of the ligand atoms coming from four L-Ser3AA-bound structures and four L-Thr3AA-bound structures clearly demonstrate a general loosening of the aminoacyl moiety upon L-Thr3AA binding (Supplementary Fig. 7a). It should also be noted here that the adenosine moiety is fairly well fixed in both L-Ser3AA- and L-Thr3AA-bound structures. This is required for maintaining the integrity of the RNA–protein interface, which is so crucial for the discriminatory mechanism. Moreover, non-overlapping error bars give substantial credence to the loosening of interactions observed and rule out any random phenomenon (Supplementary Fig. 7a).

The effect of this general loosening of interactions upon binding the cognate substrate is clearly demonstrated in Supplementary Fig. 7b,c, showing an overlap of four L-Ser3AA- and four L-Thr3AA-bound high-resolution crystal structures of ApNTD. The catalytic water Wat1 as well as the L-serine moiety is firmly fixed with the maximum displacement of 0.41 and 0.22 Å in the positions of Wat1 and Cα atom of the substrate, respectively (Supplementary Fig. 7b). On the other hand, the L-Thr3AA-bound structures of ApNTD showed the presence of Wat1 in only one out of four observations. Moreover, there was considerable displacement in the positioning of the amino-acid moiety, with the maximum displacement of 0.98 Å observed in the position of Cα atom of the substrate (Supplementary Fig. 7c). Interestingly, the L-Thr3AA-bound structure where Wat1 was observed showed a very weak electron density for the threonyl moiety, clearly indicating an incompatibility between rigid positioning of the threonyl substrate for catalysis and the presence of Wat1 (Fig. 5d). It is also worth noting here that the estimated standard uncertainties of atoms associated with L-Ser3AA-bound structures range from 0.11 to 0.16 Å, while that associated with L-Thr3AA-bound structures are from 0.13 to 0.32 Å (Supplementary Fig. 7). This obviously suggests that the difference observed in L-Ser3AA- and L-Thr3AA-bound structures is significant and not resulting from the associated coordinate errors.

### Differential remodelling of the catalytic core is the key

**Figure 6:**
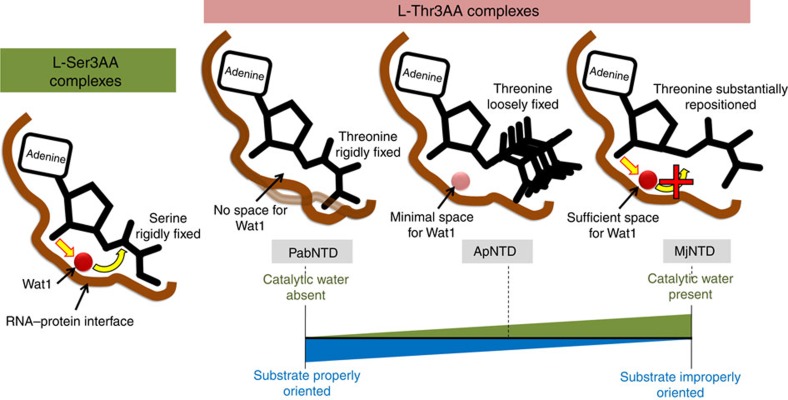
Differential remodelling of the catalytic centre for substrate discrimination. All L-Ser3AA-bound structures show a rigid positioning of the substrate along with the water Wat1 positioned at the RNA–protein interface. Accommodation of the extra γ-methyl group of threonine in the pocket has varying effects on the three NTD's studied. In PabNTD, it leads to subtle rearrangements at the interface causing a constriction of the catalytic chamber and steric exclusion of Wat1. In ApNTD, the threonyl moiety is not rigidly fixed and the space available for Wat1 is minimal, so only a weak density for Wat1 is observed if present at all. In MjNTD, the threonyl moiety is completely repositioned even though Wat1 is present in the sufficiently spacious catalytic chamber. This two-pronged approach for cognate rejection in NTD by either excluding the catalytic water from the reaction site or not allowing the substrate to be properly oriented for catalysis prevents it from mounting a nucleophilic attack on the cognate L-Thr-tRNA^Thr^ and hence no deacylation. While PabNTD and MjNTD represent two extremes, ApNTD presents an intermediate scenario.

The structural analyses suggest that the loosening of interactions observed upon L-threonine binding increases the feasibility of accommodating the catalytic water Wat1. This is highlighted by the fact that PabNTD, where Wat1 was not present in any of the six independent observations coming from three different co-crystal structures with L-Thr3AA^27^, did not show any loosening of interactions upon L-threonyl substrate binding (Supplementary Figs 6 and 8a). On the contrary, MjNTD presents a case where the catalytic water is present in each observation irrespective of the substrate, but there is a remarkable loosening of interactions upon cognate L-threonine binding leading to a complete repositioning of the substrate (Supplementary Figs 6 and 8b). ApNTD, however, presents an intermediate picture showing a subtle but definite loosening while the catalytic water is observed in just one out of four observations. More interestingly, the weak electron density observed for the threonyl moiety in the L-Thr3AA-bound structure of ApNTD, where Wat1 was present, highlights a flexibility associated with the aminoacyl part of the cognate substrate when the catalytic water is present (Fig. 5d). We also rule out the possibility of discrimination happening at the level of transition-state stabilization by modelling both non-cognate and cognate transition states in the pocket (Supplementary Fig. 9), which clearly suggests that the cognate substrate rejection happens before the nucleophilic attack. On the basis of these observations, we propose a mechanistic plasticity in cognate rejection by NTDs, where deacylation of the cognate substrate is prevented by either constricting the space required for the crucial catalytic water to sit at the reaction site or by totally repositioning the amino-acid moiety to disallow the nucleophilic attack to take place mediated by the 2′-OH of ribose (Fig. 6). The differential remodelling of the catalytic centre upon non-cognate and cognate substrate binding holds the key to discrimination in this enzyme. The remodelling observed in these cases is an outcome of packing constraints associated with cramming the extra methyl group of threonine into a pocket meant for accommodating serine. More importantly, as the structural analyses show, these packing constraints are defined by the intricate interplay of the RNA with the protein backbone. Intriguingly, despite the subtle differences in remodelling observed in the three NTD homologues (Fig. 6), they are able to perform the same biochemical function with remarkable substrate specificity. Structural studies were also carried out with the mutants of the amino-acid pocket in ApNTD. While E129A mutant also showed a loosening of interactions upon L-Thr3AA binding as observed in the case of wild type, no such loosening was observed in the case of Y115A (Supplementary Table 5; Supplementary Figs 10 and 11), thus providing a rationale for rejecting the cognate substrate in the mutants.

### Catalytic mechanism in NTD

**Figure 7:**
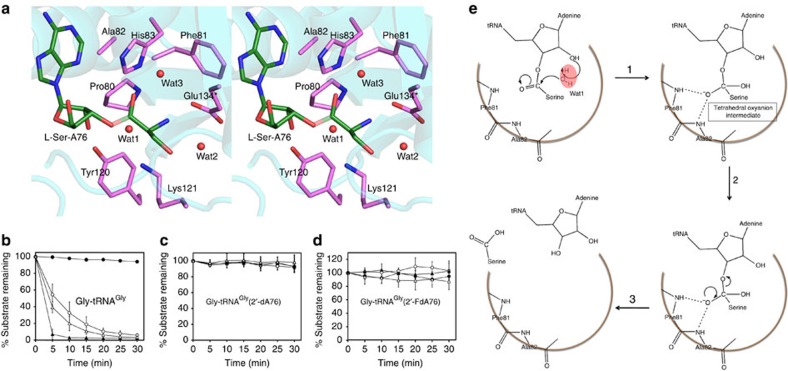
Indispensable role of RNA 2′-OH in catalysis. (**a**) Stereoscopic depiction showing all the protein side chains within 6 Å of the scissile bond (transparent) in PabNTD. Deacylation of (**b**) Gly-tRNA^Gly^, (**c**) Gly-tRNA^Gly^(2′-dA76) and (**d**) Gly-tRNA^Gly^(2′-FdA76) by buffer (closed circles), PabNTD (open circles), ApNTD (closed triangles) and MjNTD (open triangles). The error bars indicate s.d. of three independent experiments. (**e**) The catalytic mechanism in NTD involving (1) activation of water Wat1 by 2′-OH, which in turn mounts a nucleophilic attack on the carbonyl carbon of the substrate. (2) The tetrahedral transition state is stabilized by the oxyanion hole formed by main-chain nitrogens of Ala82 and Phe81 leading to (3) cleavage and release of the amino acid and the tRNA.

Although RNA-based catalysis has been indicated in NTD by our previous studies^26^,^27^, conclusive evidence in favour of the catalytic role of tRNA is still lacking. In PabNTD, there are seven side chains present within a distance of 6 Å from the scissile bond. These are Pro80, Phe81, Ala82, His83, Tyr120 and Lys121 from one monomer and Glu134 from the other monomer (Fig. 7a). Out of these, the side chains that can chemically play a catalytic role are His83, Tyr120, Lys121 and Glu134. We can rule out the role of Tyr120 and Glu134 since removing them has no effect on the deacylation activity of the enzyme (Fig. 1c,e). To test the role of His83 and Lys121 in catalysis, we mutated these residues to Ala and Met, respectively, in PabNTD and ApNTD. While the mutant H83A in PabNTD showed no activity, a moderate activity was observed in the corresponding H77A mutant in ApNTD, which argues against a catalytic role of this residue (Supplementary Fig. 12a). Moreover, the editing defect observed in these mutants could not be rescued by adding imidazole (Supplementary Fig. 12b), which further substantiates the lack of catalytic role of this residue. Importantly, this mutant in both PabNTD and ApNTD can efficiently deacylate Gly-tRNA^Gly^ (also a substrate for NTD as discussed later) clearly discarding any role of this histidine in catalysing aminoacyl-tRNA deacylation (Supplementary Fig. 12c). Significantly, this position is occupied by a Leu in DTD (Supplementary Fig. 12e), which also operates with a similar mechanistic mode^30^ thus clearly ruling out any role of this His residue in catalysis. Furthermore, H77A mutant does not show even moderate activity against L-Thr-(Ec)tRNA^Thr^, suggesting that this residue plays no role in substrate specificity as well (Supplementary Fig. 12d). On the other hand, the Lys to Met mutant, although showed partial activity against L-Ser-(Ec)tRNA^Thr^ in our earlier work^26^, in subsequent studies with not only PabNTD K121M but also the corresponding K116M and K122M mutants of ApNTD and MjNTD with multiple batches of protein showed a severely compromised activity (Supplementary Fig. 13a), suggesting a crucial role of this residue in enzyme function. However, a direct role of Lys in catalysis is still highly unlikely considering that this position is naturally occupied by a Met in the structural homologue DTD (Supplementary Fig. 13i). A catalytic role would have imposed a strict conservation of this residue in the fold. We solved the crystal structure of K121M mutant of PabNTD and K116M mutant of ApNTD with L-Ser3AA and L-Thr3AA to investigate the structural basis of the observed loss of activity (Supplementary Table 6). A significant repositioning of the crucial catalytic water was observed upon mutation of the polar Lys side chain to the non-polar Met side chain, which is present in DTD (Supplementary Fig. 13b,c). This provides a clear structural basis for the loss of function in this mutant, despite its high binding affinity for the substrate (Supplementary Fig. 13d,h). Therefore, we show that Lys plays a crucial role in positioning the water molecule for catalysis rather than acting as a nucleophile. On the basis of this evidence, a direct role of both His83 and Lys121 in catalysis can be ruled out.

### Catalytic role of 2′-OH of tRNA

We then probed the role of 2′-OH of A76 of tRNA in catalysis by using modified tRNA^Thr^(2′-dA76), which lacks the terminal 2′-OH group. However, tRNA^Thr^(2′-dA76) could not be aminoacylated by ThrRS, since the 2′-OH of A76 also plays a role in aminoacylation^31^ and therefore we could not generate the substrate for NTD using modified tRNA^Thr^. To circumvent this practical limitation, we designed an alternate strategy. Earlier work on PabNTD, based on structural and binding studies, had suggested that this enzyme would also act on glycine, which is smaller than serine^27^. Biochemical studies clearly revealed that PabNTD, ApNTD as well as MjNTD can indeed deacylate Gly-tRNA^Gly^ (Fig. 7b). Surprisingly, the activity against Gly-tRNA^Gly^ was higher than that against L-Ser-tRNA^Thr^ in all three NTDs. We then tested whether tRNA^Gly^(2′-dA76) is a substrate for aminoacylation by glycyl-tRNA synthetase (GlyRS) or not and fortuitously, GlyRS could efficiently charge tRNA^Gly^(2′-dA76) with glycine. The deacylation activity of PabNTD, ApNTD and MjNTD was then tested against Gly-tRNA^Gly^(2′-dA76). All three NTDs showed a complete loss of activity against Gly-tRNA^Gly^(2′-dA76) (Fig. 7c). We also tested the activity of all three NTDs against Gly-tRNA^Gly^(2′-FdA76), which had a fluoro group in place of –OH at the terminal 2′ position of tRNA. All three NTDs showed a complete loss of activity against Gly-tRNA^Gly^(2′-FdA76) (Fig. 7d), providing compelling evidence in support of a direct role of 2′-OH of A76 in the catalytic mechanism of NTD (Fig. 7e). The possible involvement of transacylation of the aminoacyl moiety to 2′-OH can be ruled out, since earlier studies have clearly shown that this fold acts on 3′-aminoacylated substrates^27^,^30^ and therefore the loss of activity against mutant tRNAs confirms the catalytic role of 2′-OH. Moreover, considering that RNA substrate-based catalysis has also been suggested for DTD^30^, this study clearly delineates this fold as an RNA-based catalyst.

## Discussion

**Figure 8:**
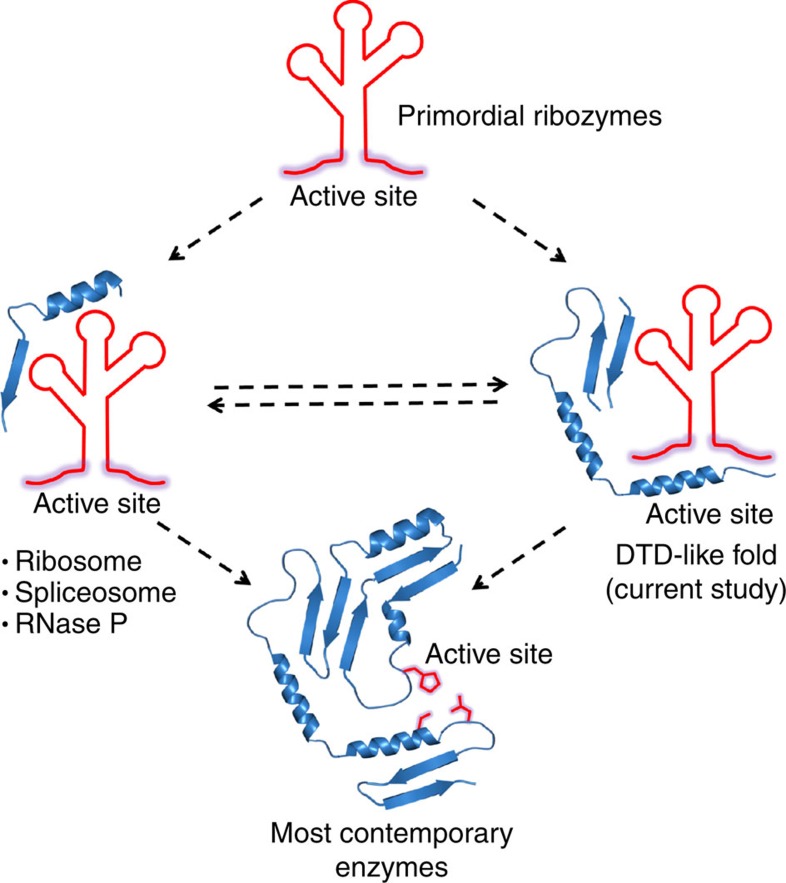
Model for transition from the RNA world to protein world. Simple peptides could have initially conjugated with primordial ribozymes for either accessory roles (for example, ribosome, spliceosome and RNase P) or for a direct functional role (DTD-like fold) in the transition phase dominated by RNA–peptide collaborations. The contemporary protein world could have emerged due to this infiltration of functional sites of early catalytic RNAs, where the chemically more diverse side chains took over the major role while RNAs were either completely removed or reduced to simple nucleotide cofactors, which are seen today in multiple functional contexts.

The role of RNA in catalysis has been noted in fundamental processes involved in the flow of genetic information such as protein synthesis by ribosome^32^, pre-mRNA splicing by spliceosome^33^ and group II introns^34^. While the role of RNA in proofreading has been suggested by some studies^35^,^36^,^37^, here we convincingly show, through extensive structural and biochemical studies, the catalytic role of RNA in proofreading by a primordial fold. Moreover, the significance of 2′-OH in proofreading shown here highlights the crucial role played by the 2′–3′ *cis*-diol chemistry of tRNA in protein synthesis, as noted earlier for several steps involved in the process^38^. Such substrate-assisted catalytic mechanisms have been proposed to be a characteristic of ancient enzymes^39^. More importantly, the study reveals a unique mechanism of substrate specificity where a subtle discrimination of a single methyl group is carried out without the role of side chains in the pocket. It is conceivable that the lack of functional role of side chains would not have allowed active-site rewiring and thereby prevented the functional expansion of this fold, providing a possible explanation as to why it is not present in multiple functional contexts across life forms. Taken together, the mode of operation presented in this study, wherein both catalysis and substrate specificity are located at the RNA–protein interface, is reminiscent of a primordial enzymatic strategy by which ancient RNA–peptide enzymes could have operated in the phase of transition from the RNA world to the protein world (Fig. 8). This is particularly interesting considering that we are still in an RNA–protein world rather than a completely protein world^39^,^40^,^41^.

Interestingly, the work elucidates the mechanistic basis of substrate discrimination by this enzyme employing differential remodelling of the catalytic centre. While active-site remodelling upon substrate binding is a relatively known phenomenon, the study reveals how an intricate interplay of RNA and protein leads to subtle differences in the way the catalytic centre is remodelled upon binding the non-cognate and cognate substrate to determine enzyme specificity. Moreover, there are notable variations in the way the catalytic centre is remodelled among different homologues while still leading to the same biochemical outcome with identical substrate specificity.

Although it is speculated that peptides could have been employed as cofactors by early ribozymes, how random peptides of the primordial era could collaborate with RNA to perform functional roles is not known^42^. The mechanistic plasticity with dispensable roles of side chains shown here suggests how the primordial RNA–peptide enzymes, with significant sequence heterogeneity and limited amino-acid repertoire, could have operated. With the addition of more amino acids with diverse side-chain chemistries and the evolution of genetic code, the chemically more competent side chains took over major enzymatic roles as observed in contemporary protein enzymes. However, this archaeal proofreading enzyme has preserved the primordial *modus operandi* probably because it makes this enzyme recalcitrant to cognate substrate deacylation, even with significant variations in amino acids lining the pocket. Moreover, considering that archaea flourish in extreme environments more reminiscent of the primordial era, reports suggesting a thermophilic origin of life^43^ and that archaeal ancestors were more primitive than other domains of life^44^, preservation of an ancient *modus operandi* in archaea seems highly plausible. Furthermore, the ability of all the tested NTDs to bind the cognate substrate without deacylating it may be an adaptation for protecting the ester linkage in aminoacyl-tRNA at high temperatures in these organisms. It is also plausible that these modules could have primarily originated for protecting the aminoacyl-tRNA esters in the harsh primordial environment, especially in a pre-elongation factor era. In any case, the study opens up some intriguing questions regarding evolution as to why certain residues, which are totally dispensable for enzyme function, are so strictly conserved and whether such a mode of operation exists in any other fold in archaea or other domains of life.

## Methods

### Cloning, expression and protein purification

The PCR-amplified gene fragments encoding 1-143, 1-136 and 5-143 residues of ThrRS from *P. abyssi*, *A. pernix* and *M. jannaschii*, respectively, and full-length *P. abyssi* ThrRS were inserted between NdeI and XhoI sites of pET-21b vector, while the full-length ApThrRS-2 gene fragment was inserted between NdeI and HindIII sites of pET-21b. An in-frame stop codon was introduced in the reverse primer to generate untagged constructs. The recombinant plasmid with the gene of interest was transformed into *E. coli* BL21(DE3) and pLysS strains in the case of full-length PabNTD and PabThrRS, respectively, and into the *E. coli* BL21-CodonPlus(DE3)-RIL strain in the case of ApNTD, ApThrRS-2 and MjNTD for overexpression. PabNTD and MjNTD were purified by a three-step procedure including anion-exchange, hydrophobic interaction (HIC) and size-exclusion (SEC) chromatographic steps. ApNTD, on the other hand, was purified using only cation-exchange chromatography followed by SEC. PabThrRS full-length and ApThrRS-2 were purified in two steps—Ni-NTA affinity chromatography followed by SEC. During anion-exchange chromatography, the supernatant was loaded onto Q Sepharose column (Amersham Pharmacia) pre-equilibrated with 50 mM Tris pH 8.0 and 20 mM NaCl, whereas for cation-exchange chromatography the supernatant was loaded onto a Sulfopropyl-Sepharose column (Amersham Pharmacia) pre-equilibrated with 50 mM Tris pH 7.0 and 20 mM NaCl. The protein of interest was eluted in a linear gradient of NaCl from 20 to 250 mM. Wherever HIC was required, the protein of interest was first treated with 30% ammonium sulphate at 4 °C for 4–5 h, followed by centrifugation at 18,000 r.p.m. for 1 h. The supernatant was then subjected to HIC by loading onto a Phenyl-Sepharose column (Amersham Pharmacia) equilibrated with 30% ammonium sulphate and 50 mM Tris pH 8.0, and eluted in a linear gradient from 30 to 0% ammonium sulphate. In the case of Ni-NTA, the supernatant was loaded onto the column pre-equilibrated with 50 mM Tris pH 8.0, 150 mM NaCl and 10 mM imidazole, followed by three washes with buffers containing 30, 50 and 100 mM imidazole. The protein was eluted with elution buffer containing 250 mM imidazole. Full-length PabThrRS was purified to homogeneity by SEC using Superdex-200 (Amersham Pharmacia), whereas all the rest of the proteins were purified to homogeneity by SEC using the Superdex-75 column (Amersham Pharmacia) equilibrated with 100 mM Tris pH 7.5 and 300 mM NaCl. The concentration of final protein was estimated by its OD_280_ measured using the NanoDrop 1,000 Spectrophotometer (Thermo Scientific). The purified protein was either used for crystallization or stored for biochemical studies. To store the protein, equal volume of 100% glycerol was added and then the protein was divided into aliquots of 100 μl each before storing them at −30 °C. For crystallization, PabNTD was buffer exchanged into 20 mM Tris pH 8.0, MjNTD was buffer exchanged into 50 mM Tris pH 8.0 and 150 mM NaCl and ApNTD was buffer exchanged into 20 mM Tris pH 8.0 before setting up the crystallization experiment. All mutants were generated using the QuickChange XL site-directed mutagenesis kit (Stratagene), and the proteins were purified by the same protocol as for the wild type.

### Crystallization and structure determination

The nonhydrolyzable analogues L-Ser3AA and L-Thr3AA were obtained from Jena Biosciences. The protein and ligand were mixed in a molar ratio of 1:5 to 1:10 and the premix was stored overnight at 4 °C before setting up the crystallization experiments by mixing 2 μl of reservoir buffer with 2 μl of premix at 4 °C. The crystallization condition for ApNTD was as reported earlier^45^, while MjNTD+L-Ser3AA crystals were obtained using 0.1 M Tris HCl pH 8.5, 0.2 M MgCl_2_ and 25% PEG3350 and MjNTD+L-Thr3AA crystals were obtained using 0.1 M Tris HCl pH 7.5, 0.2 M MgCl_2_ and 25% PEG3350 at 4 °C. PabNTD K121M crystals were obtained as the wild type earlier^27^. All diffraction data were collected at the in-house X-ray facility comprising of the RigakuMicromax007 HF rotating-anode generator with MAR345dtb image plate detector from MAR Research and FR-E+ SuperBright X-ray generator from Rigaku equipped with VariMax HF optic and R-AXIS IV++ image plate detector. The crystals were mounted on a nylon loop and flash frozen by immediately dipping in liquid nitrogen before placing them in the nitrogen gas stream at 100 K for cryocrystallographic data collection. Flash freezing of crystals led to a significant improvement in diffraction quality. However, no cryoprotectant was required, although care was taken to remove excess mother liquor from the loop before flash freezing to limit the formation of ice. All data were processed using HKL2000 (ref. 46), and the structures were solved by molecular replacement using MOLREP-AUTO MR from CCP4 suite^47^ with PabNTD apo structure (PDB ID: 1Y2Q) as the search model. The structure refinements were done using CNS^48^ and REFMAC^49^, while COOT^50^ was used for model building. The restraints for ligand refinement were obtained from PRODRG server^51^ and the structures were validated by PROCHECK^52^. Figure preparations were done in PyMOL^53^ and the catalytic chamber volumes were calculated using the high-performance computational capabilities of the Helix Systems at the National Institutes of Health, Bethesda, MD (http://helix.nih.gov).

### Biochemical assays

*E. coli* tRNA^Thr^ and tRNA^Gly^ as well as *A. pernix* tRNA^Thr^ were transcribed *in vitro* using Ambion MEGAshortscript. The radiolabelled compounds were supplied by BRIT-Jonaki, Hyderabad and MP Biomedicals. The tRNAs were 3′-end labelled with [α-^32^P]-ATP in the presence of CCA-adding enzyme using the standard procedure^54^. Aminoacylation of (Ec)tRNA^Thr^ and tRNA^Gly^ was done using a purified *E. coli* ThrRS fragment (243-642) and *Thermus thermophilus* GlyRS, respectively, whereas aminoacylation of (Ap)tRNA^Thr^ was performed with purified *P. abyssi* ThrRS K121M (editing-defective mutant). (Ec)tRNA^Thr^ was incubated at 37 °C for 10 min in a reaction containing 50 mM Tris pH 7.5, 30 mM KCl, 10 mM MgCl_2_, 5 mM dithiothreitol (DTT), 2 mM adenosine triphosphate (ATP), 2 mM amino acid (L-serine or L-threonine), 0.4 μM tRNA, 1 U ml^−1^ pyrophosphatase and 2 μM *E. coli* ThrRS fragment (243-642). tRNA^Gly^ was incubated at 37 °C for 15 min in a reaction containing 100 mM HEPES pH 7.2, 30 mM KCl, 10 mM MgCl_2_, 2 mM ATP, 50 mM glycine, 1 μM tRNA^Gly^ and 2.2 μM *T. thermophilus* GlyRS. (Ap)tRNA^Thr^ was incubated at 37 °C for 45 min in a reaction containing 100 mM HEPES pH 7.5, 10 mM KCl, 10 mM MgCl_2_, 5 mM DTT, 10 mM ATP, 200 mM L-serine or 90 mM L-threonine, 1 μM (Ap)tRNA^Thr^ and 50 μM PabThrRS K121M. Aminoacylation efficiency was ≥50% (Supplementary Fig. 14). The aminoacylated tRNAs were phenol extracted and ethanol precipitated before resuspending in 5 mM sodium acetate pH 4.6. Deacylation reactions of L-Ser-(Ec)tRNA^Thr^ or L-Thr-(Ec)tRNA^Thr^ were done by adding 15 μM of test enzyme (unless mentioned otherwise) to a reaction mixture containing 50 mM HEPES pH 7.0, 90 mM KCl, 5 mM MgCl_2_, 5 mM DTT and 0.2 μM aminoacyl-tRNA, and incubating at 37 °C for 30 min. For Gly-tRNA^Gly^ deacylation, 0.5 μM enzyme concentration was used and the reaction was carried out at 30 °C. Deacylation of L-Ser-(Ap)tRNA^Thr^ or L-Thr-(Ap)tRNA^Thr^ was also performed under the conditions mentioned above. An aliquot of reaction mix at each time point was subjected to S1 nuclease digestion for 30 min at 22 °C and analysed by thin-layer chromatography (TLC) by spotting 1 μl on a PEI cellulose sheet (Merck) as explained earlier^30^. A representative TLC run has been shown in Supplementary Fig. 15. Each point on the deacylation curves represents an average of three independent readings; the error bars indicate one s.d. from the mean value. The observed single-turnover first-order rate constant (*k*_obs_) was determined by fitting the data to the exponential decay equation *y=Ae*^*−kx*^ (refs 55, 56, 57) using GraphPad Prism software.

The use of isolated domains for biochemistry did not allow multiple-turnover or saturation kinetics to be performed, since the activity of these truncated domains was very weak at low enzyme concentrations. Moreover, biochemical studies with ApThrRS-2 also showed a generally weak activity, thereby not allowing multiple-turnover kinetics to be performed even with this enzyme.

### Deacylation assays with 2′-modified tRNA^Gly^

For generating modified tRNAs, *E. coli* tRNA^Gly^ without the terminal A76 (tRNA^Gly^-CC) was *in vitro* transcribed. Preparation of tRNA^Gly^(2′-dA76) was done by incubating 10 μM tRNA^Gly^-CC with [α-^32^P]-2′-dATP along with 2.6 μM CCA-adding enzyme at 37 °C for 4 h in a reaction containing 50 mM Tris pH 7.6, 20 mM MgCl_2_, 0.5 mM DTT and 0.6 mM CTP. For making tRNA^Gly^(2′-FdA76), first body-labelled tRNA^Gly^-CC was prepared by carrying out *in vitro* transcription in the presence of [α-^32^P]-UTP. This was followed by incorporation of 2′-fluoro-2′-deoxy-ATP(2′-FdATP) by incubating body-labelled tRNA^Gly^-CC with 400 μM 2′-FdATP from Jena Biosciences in the presence of CCA-adding enzyme, and applying the same reaction conditions as those used for 2′-dA76 incorporation. Deacylation assays with Gly-tRNA^Gly^(2′-dA76) were carried out using standard TLC-based assays. For Gly-tRNA^Gly^(2′-FdA76) deacylation, acid urea polyacrylamide gel electrophoresis was used^58^, where aliquots at each time point were added to gel loading buffer containing 100 mM sodium acetate pH 5.5, 7 M urea, 50% glycerol and 50 mg ml^−1^ each of bromophenol blue and xylene cyanol before directly loading on acid urea polyacrylamide gel electrophoresis. Each point on the deacylation curves represents an average of three independent readings; the error bars indicate one s.d. from the mean value.

### ITC binding studies

ITC-based solution binding studies were carried out using the MicroCal iTC_200_ system (GE Healthcare Life Sciences). Each titration was performed by adding 1–10 mM of ligand to 0.1 mM (in the case of ApNTD wild type and mutants) or 0.2 mM (in the case of MjNTD wild type) protein. The ligands were in 20 mM Tris pH 9.0, while the proteins were in 50 mM Tris pH 8.0 and 150 mM NaCl. The experiment was carried out at 30 °C by adding 2 μl of titrant at intervals of 4 min each. For eliminating dilution errors, titration with ligand buffer alone was performed and subtracted from the titration isotherm. Data fitting and analysis were performed using MicroCal Analysis software. *K*_d_ was calculated by applying the formula *K*_d_*=*1/*K*_a_.

## Additional information

**Accession codes:** Atomic coordinates and structure factors files have been deposited in the Protein Data Bank under accession codes 4RR6, 4RR7, 4RR8, 4RR9, 4RRA, 4RRB, 4RRC, 4RRD, 4RRF, 4RRG, 4RRH, 4RRI, 4RRJ, 4RRK, 4RRL, 4RRM, 4RRQ and 4RRR.

**How to cite this article:** Ahmed, S. *et al.* Specificity and catalysis hardwired at the RNA–protein interface in a translational proofreading enzyme. *Nat. Commun.* 6:7552 doi: 10.1038/ncomms8552 (2015).

## Supplementary Material

Supplementary Information Supplementary Figures 1-15, Supplementary Tables 1-6 and Supplementary References

## Figures and Tables

**Table 1:** Observed first-order rate constant (*k*_obs_) for deacylation of *A. pernix* L-Ser-tRNA^Thr^ by editing enzymes.

**Enzyme (1.5 μM)**	**Substrate (200 nM)**	***k***_**obs**_ (**min**^**−1**^)
ApThrRS-2 wild type	L-Ser-(Ap)tRNA^Thr^	0.352±0.060
ApThrRS-2 Y115A/E129A	L-Ser-(Ap)tRNA^Thr^	0.213±0.022
PabNTD wild type	L-Ser-(Ap)tRNA^Thr^	0.209±0.026
PabNTD Y120A/E134A	L-Ser-(Ap)tRNA^Thr^	0.143±0.018
