# Supplementary material for: Specificity and catalysis hardwired at the RNA–protein interface in a translational proofreading enzyme
Source: Nat Commun. 2015 Jun 26;6:7552. doi: 10.1038/ncomms8552 (PMC4491819; doi:10.1038/ncomms8552)
Supplement: Supplementary Information — Supplementary Figures 1-15, Supplementary Tables 1-6 and Supplementary References [file ncomms8552-s1.pdf]

Supplementary information

Supplementary figures

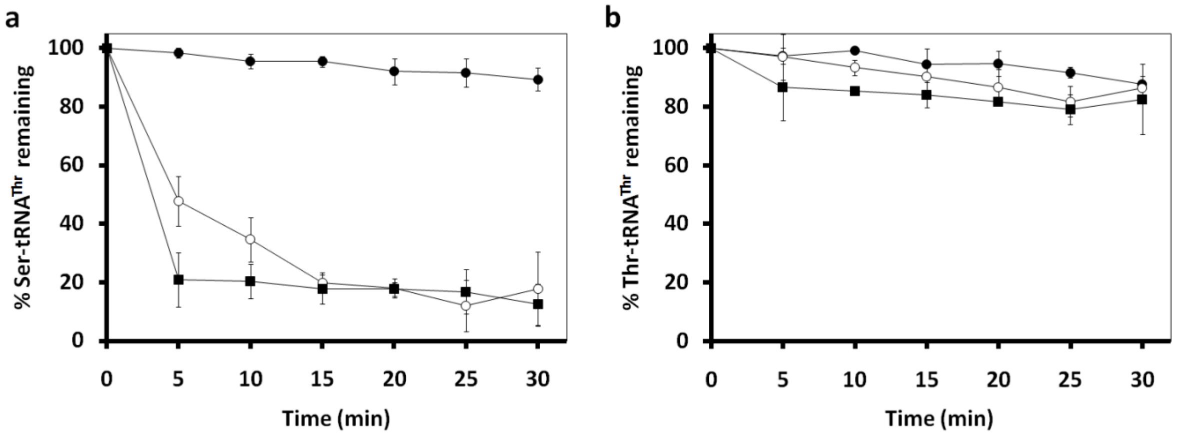

Supplementary figure 1. Deacylation of (**a**) L-Ser-(Ec)tRNA^{Thr} and (**b**) L-Thr-(Ec)tRNA^{Thr} by buffer (closed circles), PfuNTD wild type (open circles) and PfuNTD Y120A/E134A (closed squares). The error bars indicate standard deviation of three independent experiments.

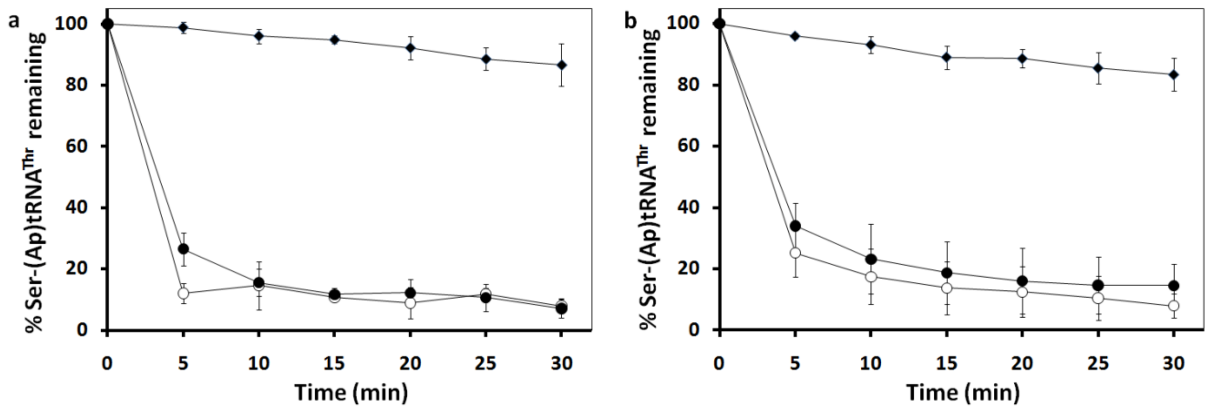

Supplementary figure 2. (**a**) Deacylation of 0.2 µM L-Ser-(Ap)tRNA^{Thr} by buffer (closed diamond), 1.5 µM ApThrRS-2 wild type (open circle) and 1.5 µM ApThrRS-2 Y115A/E129A (closed circle). (**b**) Deacylation of 0.2 µM L-Ser-(Ap)tRNA^{Thr} by buffer (closed diamond), 1.5 µM PabNTD wild type (open circle) and 1.5 µM PabNTD Y120A/E134A (closed circle). The error bars indicate standard deviation of three independent experiments.

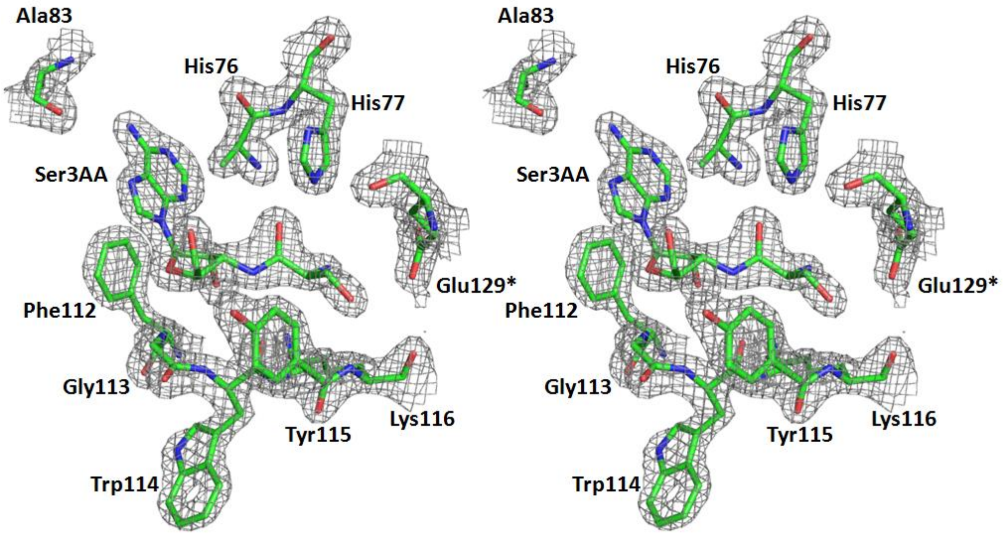

Supplementary figure 3. Stereoscopic representation of a portion of ApNTD+L-Ser3AA structure showing $2F_o$-F_c electron density map contoured at 1.5σ.

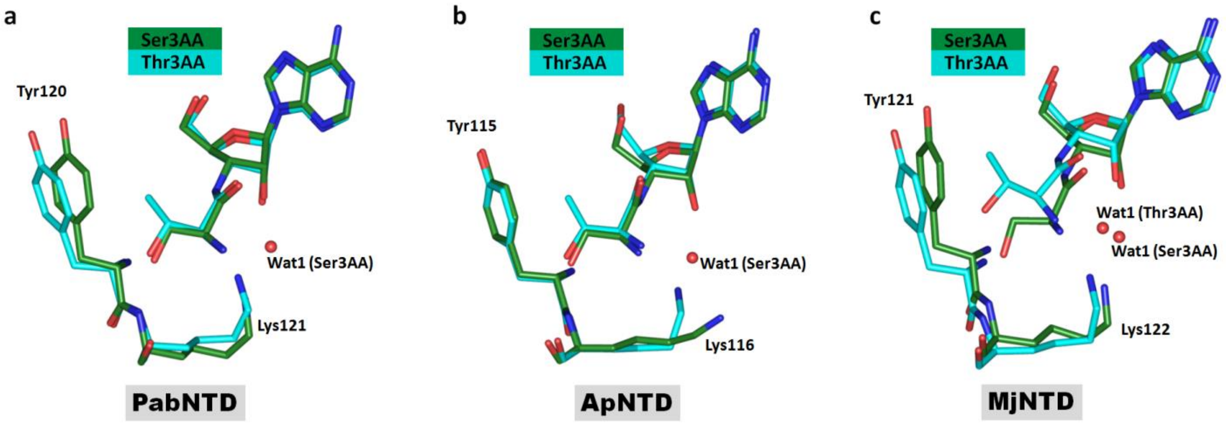

Supplementary figure 4. Superimposition of L-Ser3AA- (green) and L-Thr3AA-bound (cyan) structures in (**a**) PabNTD (**b**) ApNTD and (**c**) MjNTD. The proposed cognate rejection mechanism based on PabNTD structures had implicated the repositioning of Tyr120 side chain upon accommodation of extra γ-methyl group of threonine and a concomitant movement of adjacent Lys121 leading to steric exclusion of putative catalytic water Wat1[1].

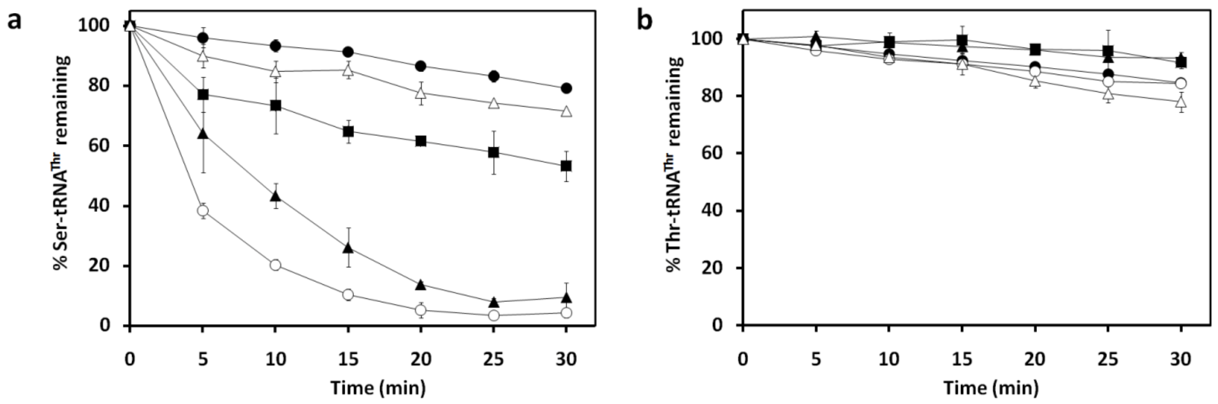

Supplementary figure 5. Deacylation of (**a**) L-Ser-(Ec)tRNA^{Thr} and (**b**) L-Thr-(Ec)tRNA^{Thr} by buffer (closed circles), MjNTD wild type (open circles), Y121A (closed triangles), E135A (open triangles) and Y121A/E135A (closed squares). The error bars indicate standard deviation of three independent experiments.

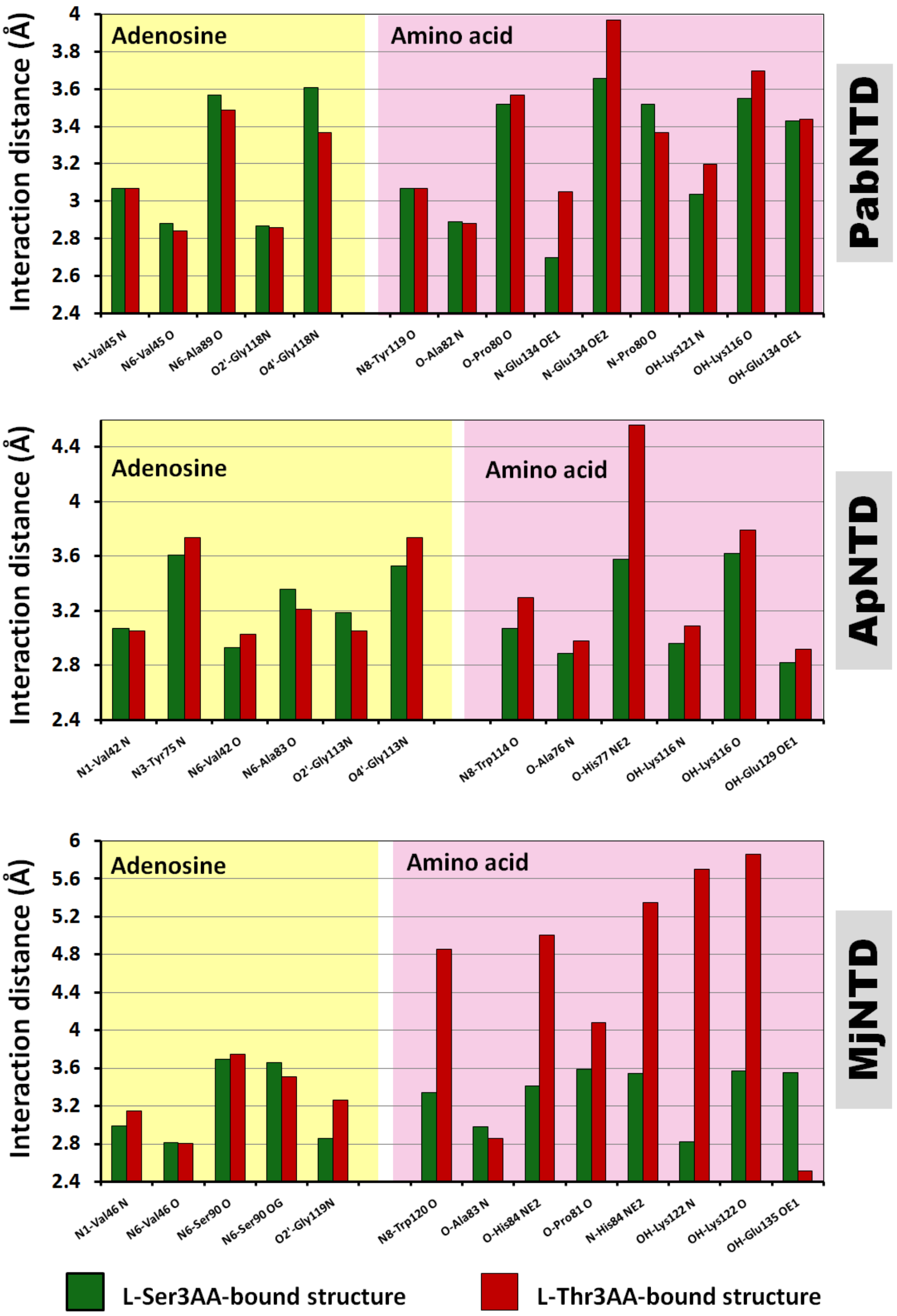

Supplementary figure 6. Interaction distances of the ligand in the pocket. Interactions of L-Ser3AA atoms are represented by green bars and that of L-Thr3AA atoms by red bars. The atoms belonging to adenosine moiety are in the yellow region while the atoms from amino acid moiety are in the pink region.

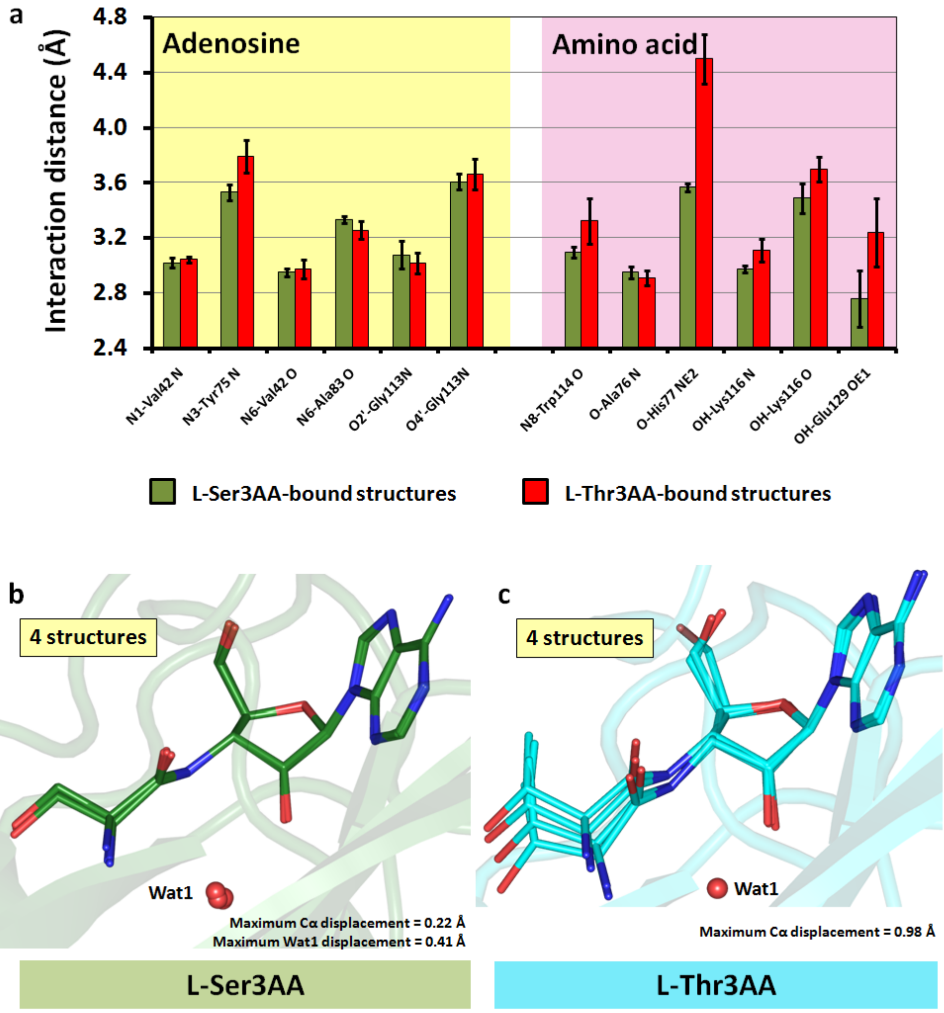

	ApNTD+L-Ser3AA	ApNTD+L-Thr3AA
Resolution (Å) of all crystal structures	1.67-1.88	1.70-2.10
Estimated standard uncertainties (Å) for all structures	0.11-0.16	0.13-0.32

Supplementary figure 7. (**a**) The average interaction distance of ligand atoms in multiple crystal structures of ApNTD. L-Ser3AA atoms are represented in green bars and L-Thr3AA atoms in red bars. The atoms belonging to adenosine moiety are in the yellow region while the atoms from amino acid moiety are in the pink region. The L-Thr3AA-bound structures show a general loosening of interactions of the aminoacyl moiety compared to L-Ser3AA-bound structures. Overlap of 4 crystal structures of ApNTD in complex with (**b**) L-Ser3AA and (**c**) L-Thr3AA. The maximum displacement in Cα of L-Ser3AA and Wat1 are 0.22 Å and 0.41 Å respectively in L-Ser3AA-bound structures. In L-Thr3AA-bound structures, the maximum displacement in Cα of L-Thr3AA is 0.98 Å while Wat1 is observed in only one structure.

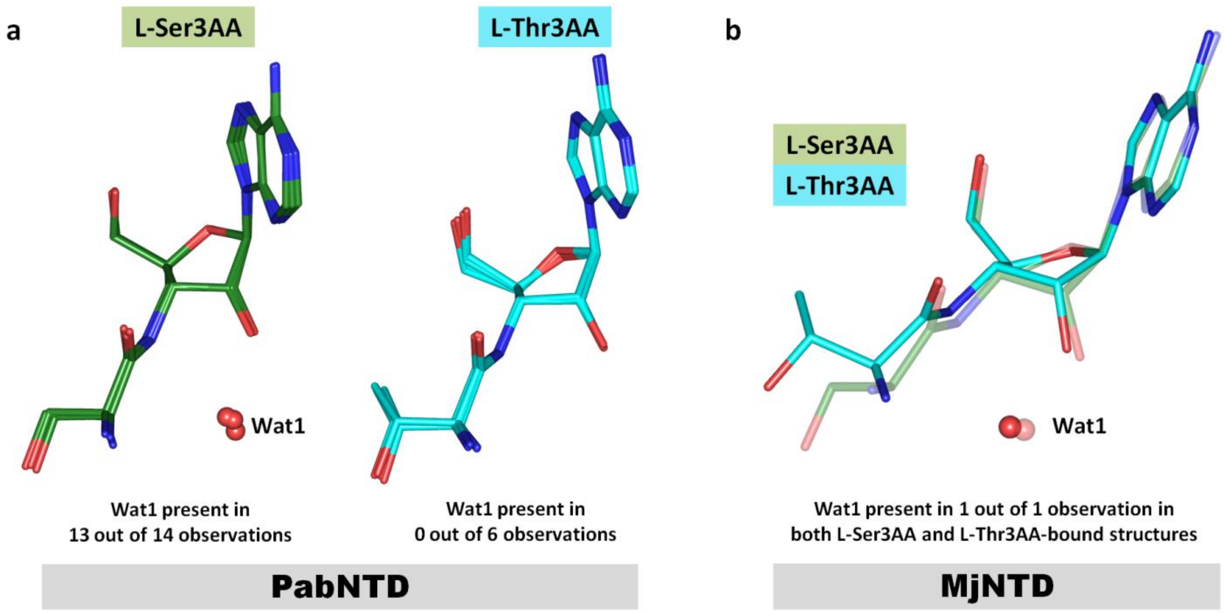

Supplementary figure 8. (**a**) Overlap of L-Ser3AA-bound structures (green) and L-Thr3AA-bound structures (cyan) of PabNTD. Both L-Ser3AA and L-Thr3AA are rigidly fixed without any significant deviation. Wat1 is present in 13 out of 14 observations of L-Ser3AA and 0 out of 6 observations of L-Thr3AA. (**b**) Overlap of L-Ser3AA-bound structures (green and transparent) and L-Thr3AA-bound structures (cyan) of MjNTD. While Wat1 is present in both structures, there is a significant repositioning of the threonyl moiety with respect to the seryl moiety.

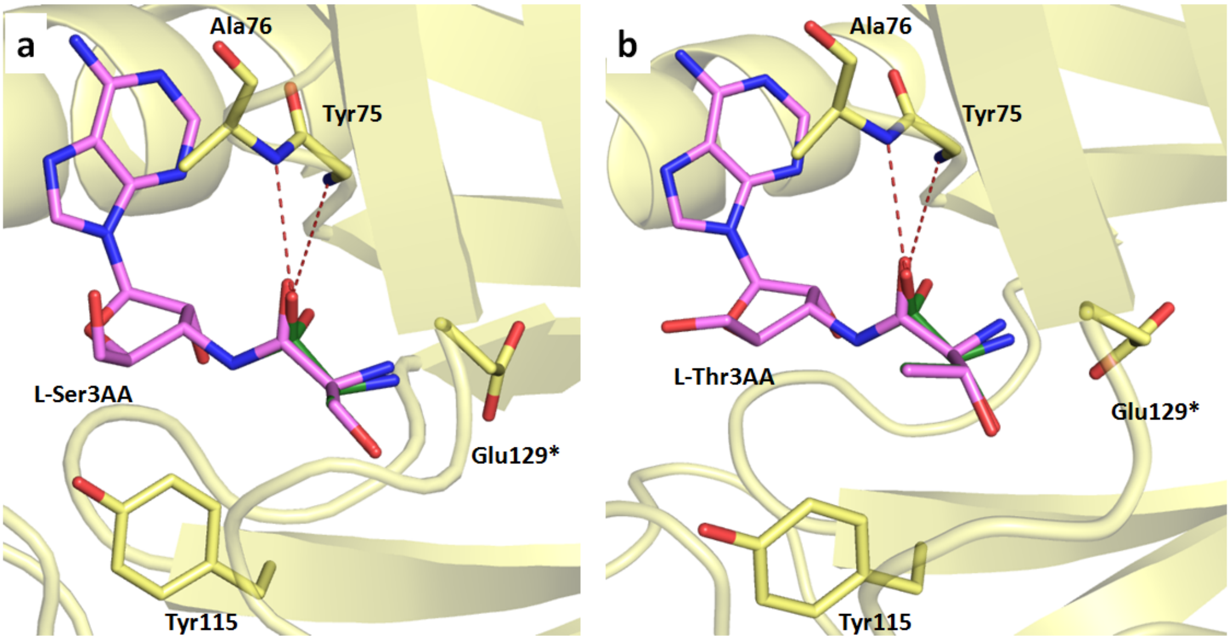

Supplementary figure 9. Modeling the oxyanion transition state (green) of (**a**) L-Ser-adenosine ester and (**b**) L-Thr-adenosine ester on the corresponding substrate analogs (pink) in the active site of ApNTD does not show any incompatibility in the case of L-Thr-adenosine ester.

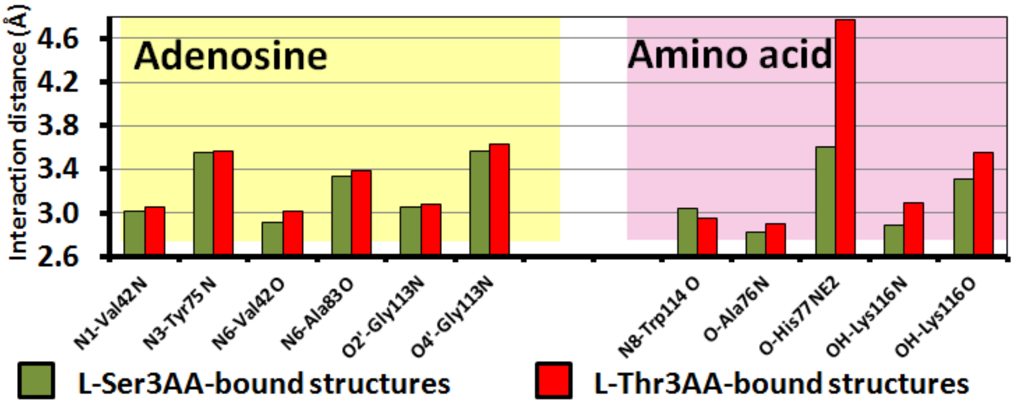

Supplementary figure 10. The interaction distances show a loosening of interactions of the threonyl moiety as observed earlier for the wild type.

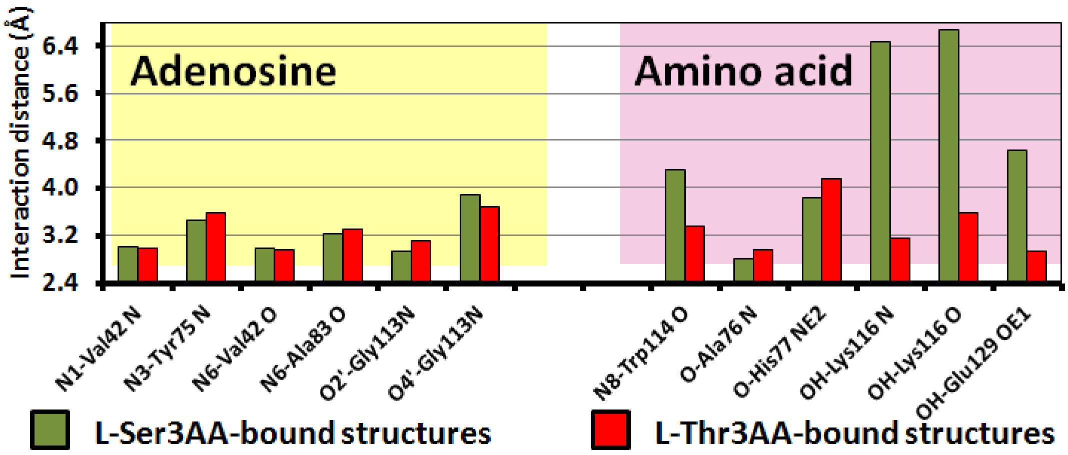

Supplementary figure 11. The interaction distances do not show any loosening of interactions of the threonyl moiety. Some interactions in the L-Ser3AA-bound structures are broken because the β-OH group of L-Ser3AA is oriented differently from that of L-Thr3AA.

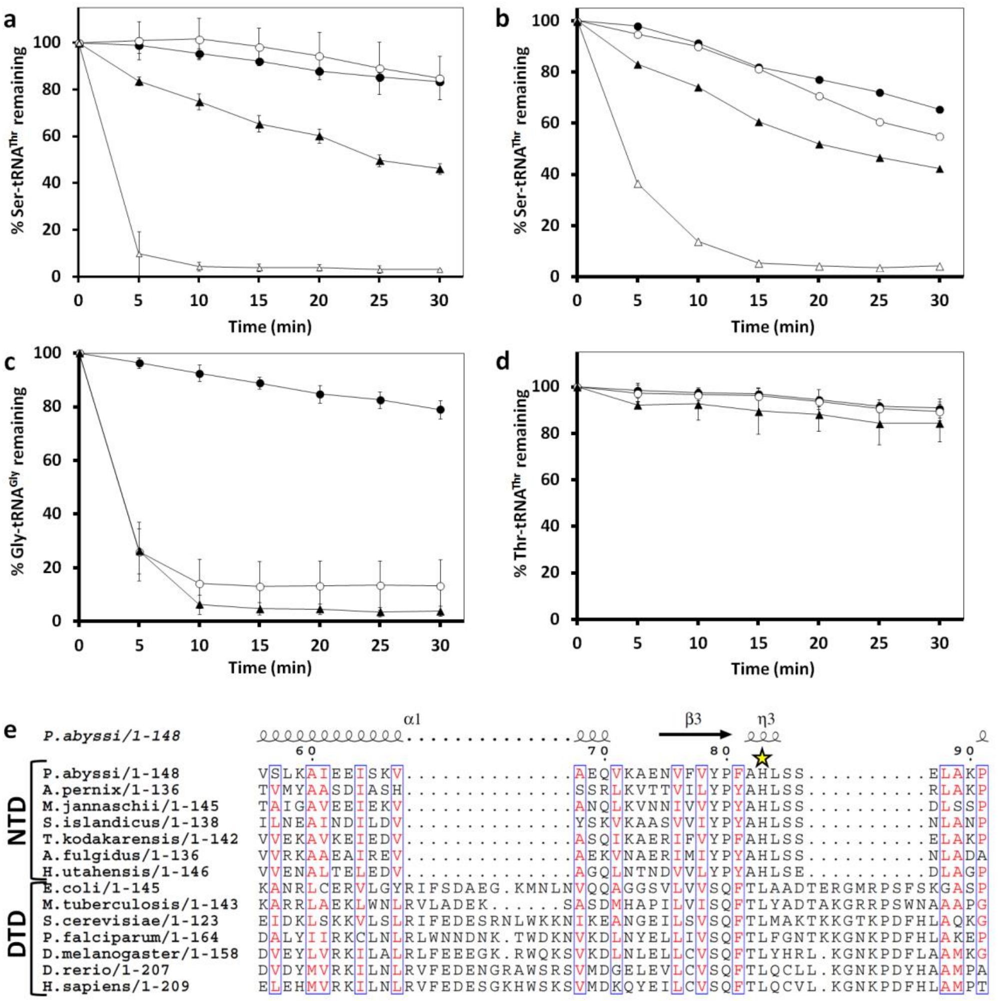

Supplementary figure 12. Evidence against the role of His83 in catalysis or substrate specificity. (**a**) Deacylation of L-Ser-(Ec)tRNA^{Thr}, (**b**) Imidazole rescue assay in the presence of 0.1 M imidazole pH 7.0, (**c**) Deacylation of Gly-tRNA^{Gly}, (**d**) Deacylation of L-Thr-(Ec)tRNA^{Thr}. The representations used in all the plots are: buffer (closed circles), PabNTD H83A (open circles), ApNTD H77A (closed triangles) and ApNTD wild type (open triangles). The error bars indicate standard deviation of three independent experiments. (**e**) Structure-based multiple sequence alignment showing that His83 (marked by yellow star) is not conserved across the DTD-like fold. All deacylation experiments were done in triplicates except imidazole rescue assay which was done once.

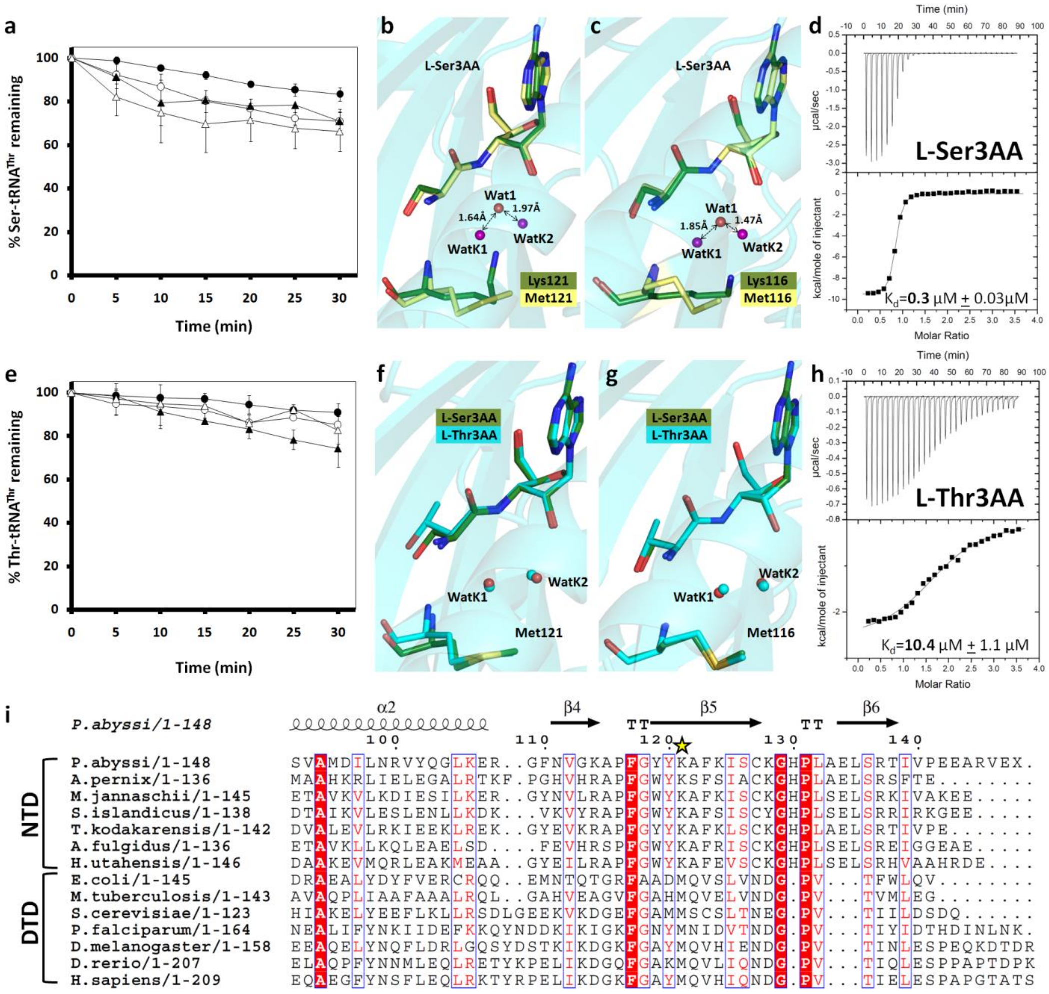

Supplementary figure 13. Evidence against the role of Lys121 in catalysis or substrate specificity. Deacylation of (**a**) L-Ser-(Ec)tRNA^{Thr} and (**e**) L-Thr-(Ec)tRNA^{Thr} by buffer (closed circles), PabNTD K121M (open circles), ApNTD K116M (closed triangles) and MjNTD K122M (open triangles). The error bars indicate standard deviation of three independent experiments. Structural overlap of wild type and K to M mutants of (**b**) PabNTD and (**c**) ApNTD in complex with L-Ser3AA. The waters WatK1 and WatK2 (purple) in K to M mutants are significantly repositioned compared to Wat1 (red) in the wild type. The ITC binding studies of PabK121M with (**d**) L-Ser3AA and (**h**) L-Thr3AA show a ~10-fold and ~3-fold increase in the affinity for L-Ser3AA and L-Thr3AA respectively compared to the wild type. Structural overlap of L-Ser3AA (green) and L-Thr3AA (cyan) complexes of (**f**) PabNTD K121M and (**g**) ApNTD K116M shows a similar repositioning of the Waters in the reaction site providing the mechanistic basis for lack of deacylation activity against both L-Ser-tRNA^{Thr} and L-Thr-tRNA^{Thr}. (**i**) Structure-based multiple sequence alignment shows that the Lys (marked by a yellow star) is replaced by a Met in DTD.

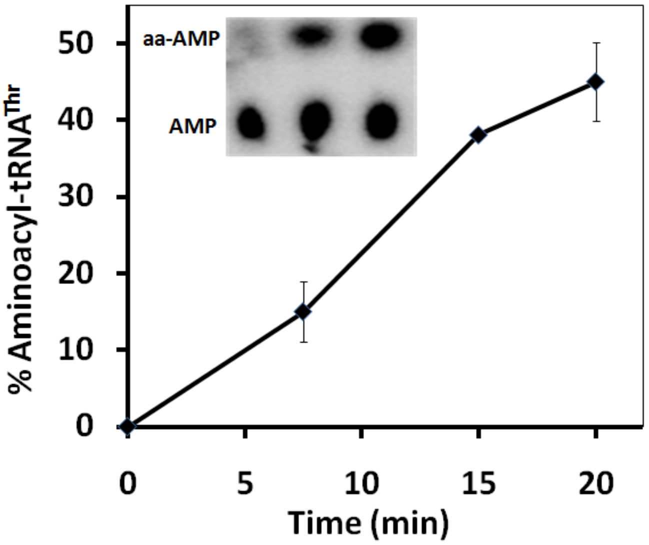

Supplementary figure 14. Percentage aminoacylation of (Ap)tRNA^{Thr} with respect to time, along with a representative TLC after S1 digestion. The error bars indicate standard deviation of three independent experiments.

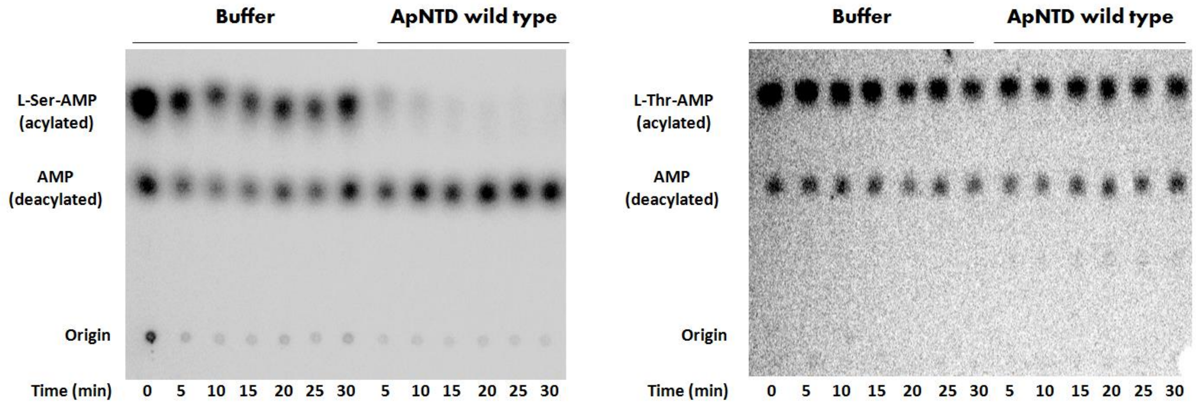

Supplementary figure 15. A representative TLC run showing aminoacyl-AMP and AMP after S1 nuclease digestion. Deacylation of L-Ser-tRNA^{Thr} and L-Thr-tRNA^{Thr} with ApNTD wild type. Substrate preparations contain at least 50% aminoacylated tRNA.

Supplementary tables

Supplementary table 1. All known mutations in proofreading domains that lead to deacylation of cognate aminoacyl-tRNA.

**Mutation(s)**	**Editing domain**	**Organism**	**Site of mutation**	**Reference**
T233A<br>H319A	IleRS	*Thermus thermophilus*	Amino acid binding pocket	*(2)*
T252A	LeuRS	*Escherichia coli*	Amino acid binding pocket	*(3)*
H369A<br>H369C	ProRS INS domain	*Escherichia coli*	Putative amino acid binding pocket	*(4)*
L202A<br>S211A<br>D234A<br>T236A	PheRS	*Pyrococcus horikoshii*	Amino acid binding pocket	*(5)*
Q633M	AlaRS	*Pyrococcus horikoshii*	Amino acid binding pocket	*(6)*
Q584N	AlaRS	*Escherichia coli*	Amino acid binding pocket	*(7)*
T30V	AlaX<br>(*trans* editing domain)	*Pyrococcus horikoshii*	Amino acid binding pocket	*(6)*

Supplementary table 2. Data collection and refinement statistics.

	**ApNTD+ L-Ser3AA**	**ApNTD+ L-Thr3AA**	**MjNTD+ L-Ser3AA**	**MjNTD+ L-Thr3AA**
**Data collection**				
Space group	$P4_12_12$	$P4_12_12$	$C2$	$P2_1$
Cell dimensions				
a, *b*, *c* (Å)	46.9, 46.9, 112.7	47.3, 47.3, 112.4	159.6, 52.8,98.3	36.6, 64.9, 97.8
α, β, γ (°)	90.0, 90.0, 90.0	90.0, 90.0, 90.0	90.0, 104.0, 90.0	90.0, 91.1, 90.0
Resolution (Å)	25.0-1.88 (1.95-1.88)*	25.0-1.70 (1.76-1.70)	25.0-1.70 (1.76-1.70)	25.0-1.93 (2.00-1.93)
R_{merge} (%)	7.4 (60.5)	6.5 (42.3)	14.7 (63.4)	5.5 (19.1)
$I / \sigma I$	21.7(2.2)	38.3 (5.3)	12.0 (2.3)	36.1 (7.6)
Completeness (%)	100 (100)	96.4 (99.8)	91.0 (99.0)	99.3 (93.1)
Redundancy	7.3 (6.8)	10.6 (11.1)	4.7 (4.6)	6.5 (5.8)
**Refinement**				
Resolution (Å)	1.88	1.70	1.70	1.93
No. reflections	10328	13567	75775	32739
R_{work} / R_{free}**	0.18/0.22	0.20/0.26	0.19/0.24	0.19/0.23
No. atoms	1246	1218	7221	4724
Protein	1068	1068	6591	4363
Ligand/ion	25/0	26/1	150/3	78/0
Water	153	123	477	283
B-factors (Å^2)	27.9	26.7	27.1	29.1
Protein	25.9	25.3	26.4	28.2
Ligand/ion	21.7/ –	28.7/35.1	27.4/30.5	33.3/ –
Water	42.5	38.2	36.9	40.4
R.m.s. deviations				
Bond lengths (Å)	0.016	0.016	0.015	0.009
Bond angles (°)	1.621	1.864	1.782	1.185

*Values in parentheses are for highest-resolution shell

**Throughout the refinement, 5% of the total reflections were held aside for R_{free}

Supplementary table 3. Data collection and refinement statistics.

	**ApNTD+ L-Ser3AA2**	**ApNTD+ L-Ser3AA3**	**ApNTD+ L-Ser3AA4**
**Data collection**			
Space group	$P4_12_12$	$P4_12_12$	$P4_12_12$
Cell dimensions			
a, *b*, *c* (Å)	47.9, 47.9, 114.7	47.8, 47.8, 114.4	47.9, 47.9,113.7
α, β, γ (°)	90.0, 90.0, 90.0	90.0, 90.0, 90.0	90.0, 90.0, 90.0
Resolution (Å)	25.0-1.86 (1.93-1.86)*	25.0-1.86 (1.93-1.86)	25.0-1.66 (1.72-1.66)
R_{merge} (%)	4.4 (28.1)	11.0 (44.2)	6.0 (31.2)
$I / \sigma I$	56.6 (6.9)	28.5(3.5)	47.8 (4.7)
Completeness (%)	98.6 (95.7)	99.7 (96.6)	98.6 (85.7)
Redundancy	13.5 (12.7)	19.2 (13.7)	12.5 (8.3)
**Refinement**			
Resolution (Å)	1.86	1.86	1.67
No. reflections	11129	11182	15270
R_{work} / R_{free}**	0.19/0.24	0.18/0.23	0.19/0.22
No. atoms	1260	1264	1267
Protein	1068	1068	1068
Ligand/ion	25/1	25/1	25/1
Water	166	170	173
B-factors (Å^2)	27.4	27.6	26.3
Protein	26.1	26.2	24.5
Ligand/ion	22.8/67.1	27.6/46.8	25.3/32.6
Water	40.8	41.2	38.9
R.m.s. deviations			
Bond lengths (Å)	0.013	0.013	0.017
Bond angles (°)	1.654	1.633	1.965

*Values in parentheses are for highest-resolution shell

**Throughout the refinement, 5% of the total reflections were held aside for R_{free}

Supplementary table 4. Data collection and refinement statistics.

	ApNTD+ L-Thr3AA2	ApNTD+ L-Thr3AA3	ApNTD+ L-Thr3AA4
**Data collection**			
Space group	$P4_12_12$	$P4_12_12$	$P4_12_12$
Cell dimensions			
a, *b*, *c* (Å)	47.1, 47.1, 112.7	48.8, 48.8, 114.7	47.5, 47.5,113.6
α, β, γ (°)	90.0, 90.0, 90.0	90.0, 90.0, 90.0	90.0, 90.0, 90.0
Resolution (Å)	25.0-2.10 (2.18-2.10)*	25.0-1.86 (1.93-1.86)	25.0-1.86 (1.93-1.86)
R_{merge} (%)	6.3 (34.8)	4.6 (21.0)	4.6 (33.2)
$I / \sigma I$	19.6 (3.3)	52.9(9.7)	50.8 (5.0)
Completeness (%)	94.0 (97.5)	99.8 (99.5)	99.8 (97.6)
Redundancy	6.3 (5.6)	13.3 (12.8)	12.8 (11.4)
**Refinement**			
Resolution (Å)	2.10	1.86	1.86
No. reflections	7137	11646	11005
R_{work} / R_{free}**	0.21/0.27	0.19/0.25	0.18/0.22
No. atoms	1177	1315	1287
Protein	1068	1068	1068
Ligand/ion	26/0	26/0	26/1
Water	83	221	192
B-factors ($Å^2$)	32.6	22.9	27.2
Protein	32.0	20.2	25.0
Ligand/ion	34.3/ –	24.9/ –	30.6/32.1
Water	40.1	35.9	39.1
R.m.s. deviations			
Bond lengths (Å)	0.012	0.011	0.017
Bond angles (°)	1.432	1.459	1.924

*Values in parentheses are for highest-resolution shell

**Throughout the refinement, 5% of the total reflections were held aside for R_{free}

Supplementary table 5. Data collection and refinement statistics.

	**ApNTD E129A+ L-Ser3AA**	**ApNTD E129A+ L-Thr3AA**	**ApNTD Y115A+ L-Ser3AA**	**ApNTD Y115A+ L-Thr3AA**
**Data collection**				
Space group	$P4_12_12$	$P4_12_12$	$P4_12_12$	$P4_12_12$
Cell dimensions				
a, *b*, *c* (Å)	47.0, 47.0, 112.5	47.1, 47.1, 112.7	47.7, 47.7,113.1	47.9,47.9,114.4
α, β, γ (°)	90.0, 90.0, 90.0	90.0, 90.0, 90.0	90.0, 90.0, 90.0	90.0, 90.0, 90.0
Resolution (Å)	25.0-1.96 (2.03-1.96)*	25.0-1.55 (1.61-1.55)	25.0-1.86 (1.93-1.86)	25.0-1.86 (1.93-1.86)
R_{merge} (%)	7.0 (57.0)	9.2 (26.3)	12.5 (73.1)	4.3 (20.9)
$I / \sigma I$	23.2 (2.0)	32.4 (7.3)	13.6 (2.1)	49.1 (8.9)
Completeness (%)	97.4 (87.8)	99.3 (96.4)	99.2 (99.7)	99.7 (98.3)
Redundancy	6.8 (5.8)	13.9 (7.5)	12.8 (11.4)	13.9 (12.6)
**Refinement**				
Resolution (Å)	1.97	1.55	1.86	1.86
No. reflections	8950	18120	10905	11276
R_{work} / R_{free}**	0.18/0.24	0.19/0.26	0.19/0.23	0.18/0.22
No. atoms	1161	1238	1230	1215
Protein	1060	1064	1061	1061
Ligand/ion	25/0	26/2	25/0	26/1
Water	76	146	144	127
B-factors (Å^2)	36.4	20.9	35.5	21.4
Protein	35.8	19.3	34.1	20.1
Ligand/ion	29.3/ −	16.9/25.2	35.1/ −	19.9/26.0
Water	46.4	33.5	46.4	32.7
R.m.s. deviations				
Bond lengths (Å)	0.016	0.015	0.013	0.010
Bond angles (°)	1.656	1.744	1.717	1.409

*Values in parentheses are for highest-resolution shell

**Throughout the refinement, 5% of the total reflections were held aside for R_{free}

Supplementary table 6. Data collection and refinement statistics.

	PabNTD K121M+ L-Ser3AA	PabNTD K121M+ L-Thr3AA	ApNTD K116M+ L-Ser3AA	ApNTD K116M+ L-Thr3AA
**Data collection**				
Space group	$P2_12_12_1$	$P2_12_12_1$	$P4_12_12$	$P4_12_12$
Cell dimensions				
a, *b*, *c* (Å)	40.6, 74.6, 96.3	38.6, 66.3, 93.7	47.2, 47.2,113.0	47.1,47.1,113.1
α, β, γ (°)	90.0, 90.0, 90.0	90.0, 90.0, 90.0	90.0, 90.0, 90.0	90.0, 90.0, 90.0
Resolution (Å)	25.0 – 1.79 (1.85 - 1.79)*	25.0 – 1.86 (1.93 - 1.86)	25.0-1.55 (1.61-1.55)	25.0-1.50 (1.55-1.50)
R_{merge} (%)	4.0 (17.4)	6.9 (17.1)	6.8 (25.2)	13.6 (47.0)
$I / \sigma I$	50.0 (6.9)	26.3 (7.6)	39.2 (11.6)	20.0(4.5)
Completeness (%)	97.2 (86.1)	99.1 (96.0)	92.7 (89.5)	99.3 (98.3)
Redundancy	5.7 (5.2)	4.8 (4.5)	14.1 (16.0)	13.7 (9.0)
**Refinement**				
Resolution (Å)	1.79	1.86	1.55	1.50
No. reflections	27566	20636	17012	19968
R_{work} / R_{free}**	0.21/0.24	0.20/0.23	0.20/0.23	0.19/0.26
No. atoms	2556	2528	1212	1195
Protein	2225	2267	1067	1067
Ligand/ion	50/0	52/0	25/1	26/1
Water	281	209	119	101
B-factors (Å^2)	19.1	25.7	18.7	22.5
Protein	18.1	25.0	17.6	21.5
Ligand/ion	15.2/–	19.2/–	18.4/21.8	20.0/26.8
Water	27.4	35.0	29.0	33.2
R.m.s. deviations				
Bond lengths (Å)	0.005	0.005	0.016	0.018
Bond angles (°)	1.36	1.39	1.84	1.94

*Values in parentheses are for highest-resolution shell

**Throughout the refinement, 5% of the total reflections were held aside for R_{free}

Supplementary References

1. Hussain, T., Kamarthapu, V., Kruparani, S.P., Deshmukh, M.V. & Sankaranarayanan, R. Mechanistic insights into cognate substrate discrimination during proofreading in translation. *Proc. Natl. Acad. Sci. USA* **107**, 22117–221121 (2010).
2. Fukunaga, R. & Yokoyama, S. Structural basis for substrate recognition by the editing domain of isoleucyl-tRNA synthetase. *J. Mol. Biol.* **359**, 901–912 (2006).
3. Mursinna, R.S., Lincecum, T.L., Jr. & Martinis, S.A. A conserved threonine within Escherichia coli leucyl-tRNA synthetase prevents hydrolytic editing of leucyl-tRNALeu. *Biochemistry* **40**, 5376–5381 (2001).
4. Wong, F.C., Beuning, P.J., Nagan, M., Shiba, K. & Musier-Forsyth, K. Functional role of the prokaryotic proline-tRNA synthetase insertion domain in amino acid editing. *Biochemistry* **41**, 7108–7115 (2002).
5. Sasaki, H.M. et al. Structural and mutational studies of the amino acid-editing domain from archaeal/eukaryal phenylalanyl-tRNA synthetase. *Proc. Natl. Acad. Sci. USA* **103**, 14744–14749 (2006).
6. Sokabe, M., Okada, A., Yao, M., Nakashima, T. & Tanaka, I. Molecular basis of alanine discrimination in editing site. *Proc. Natl. Acad. Sci. USA* **102**, 11669–11674 (2005).
7. Pasman, Z. et al. Substrate specificity and catalysis by the editing active site of Alanyl-tRNA synthetase from Escherichia coli. *Biochemistry* **50**, 1474–1482 (2011).
